# Precision‐Arranged DNA Origami Plasmonic Nanoantennas for Multidimensional Smart‐Warning of Weightlessness Induced Bone Loss

**DOI:** 10.1002/advs.202507189

**Published:** 2025-08-11

**Authors:** Yufan Ling, Xuan Qin, Weijia Sun, Fan Yue, Yiwei Wang, Di Fan, Haoyuan Xu, Ruao Xie, Jiawei Zhang, Jianwei Li, Bingyan Li, Yang Yang, Yingxian Li, Houyu Wang, Guangming Zhou

**Affiliations:** ^1^ State Key Laboratory of Radiation Medicine and Protection School of Radiation Medicine and Protection Suzhou Medical College of Soochow University Suzhou 215123 P. R. China; ^2^ Suzhou Key Laboratory of Nanotechnology and Biomedicine Institute of Functional Nano & Soft Materials & Collaborative Innovation Center of Suzhou Suzhou 215123 P. R. China; ^3^ National Key Laboratory of Space Medicine China Astronaut Research and Training Center Beijing 100094 P. R. China; ^4^ Department of Nutrition and Food Hygiene School of Public Health Soochow University Suzhou 215123 P. R. China; ^5^ Department of Thoracic Surgery Shanghai Pulmonary Hospital School of Medicine Tongji University Shanghai 200433 P. R. China

**Keywords:** bone Loss, deep learning, DNA origami, sensor, spaceflight

## Abstract

Surface‐Enhanced Raman Scattering (SERS) shows promise for monitoring health during space missions, particularly in assessing the effects of microgravity and radiation. However, traditional SERS sensors struggle with precise interfacial engineering, leading to a relatively poor assembly efficiency, and are unable to meet the practical needs of extreme spaceflight environments. To address this, it is designed and fabricated precision‐arranged DNA origami plasmonic nanoantennas. By leveraging DNA origami's addressability, it is built a 3 × 4 antenna array with a controlled spacing of 21.76 nm, enhancing assembly efficiency fourfold compared to disordered systems. The ordered system enabled accurate detection of calcium ions, interleukin‐6, and microRNA‐214 in serum from mice exposed to microgravity and radiation, with intraclass correlation coefficients > 0.75, comparable to ELISA and qPCR. More importantly, integrating the system with a convolutional neural network enabled precise bone health prediction. This platform provides a promising tool for astronaut health monitoring.

## Graphical abstract

It is synthesized precision‐arranged DNA origami plasmonic nanoantennas for multiplexed and intelligent warning of bone loss induced by microgravity and radiation. The ordered nanoantennas simultaneously and accurately detected calcium ions, interleukin‐6, and microRNA‐214 in serum from mice exposed to microgravity and radiation, with performance comparable to ELISA and qPCR. Integration with a convolutional neural network enabled accurate bone loss prediction, highlighting its potential in smart monitoring of astronaut health. The image is created by BioRender (biorender.com).

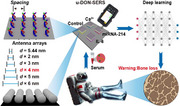

## 1 Introduction

Space exploration is entering a new era with upcoming lunar and Mars missions, and meanwhile, astronauts will increasingly face extreme conditions distinct from those of Earth.^[^ ^1^ ^]^ These include microgravity, high atomic number Z and high‐energy ions, weak magnetic fields, altered circadian rhythms, confinement, hazardous gases, and noise.^[^ ^2^, ^3^, ^4^ ^]^ Decades of spaceflight research have demonstrated that such environments negatively impact on human health and might cause muscle atrophy, bone loss, immune dysregulation, cardiovascular issues, and even cancer.^[^ ^2^, ^3^, ^4^, ^5^ ^]^ The unique conditions of spaceflight require astronaut health monitoring systems that minimize the use of space and resources.^[^ ^6^, ^7^, ^8^ ^]^ Therefore, conventional diagnostic equipment, such as computed tomography (CT), is too bulky and energy‐intensive for space use.

Alternatively, electrochemical sensors (e.g., i‐STAT), reflectance spectroscopy (e.g., the Reflotron system), and chemiluminescence devices (e.g., the PLEIADES system) are utilized at the international space station (ISS) to monitor astronaut health.^[^ ^9^, ^10^, ^11^ ^]^ However, these technologies face significant challenges, including vulnerability to interference from external environments, relatively low sensitivity, poor reliability, and/or operational complexity.^[^ ^9^ ^]^ Impressively, plasmonic nanostructures‐based surface‐enhanced Raman scattering (SERS) technique excels in environmental and body fluid detection, providing high sensitivity, specificity, rapid response, and non‐destructive analysis in a wide range of sample types.^[^ ^12^, ^13^, ^14^, ^15^ ^]^ Yet, these SERS sensors often fail to achieve precise interfacial engineering, leading to a relatively low assembly efficiency, suboptimal thermodynamics, and poor reproducibility even when the hotspots are homogeneous.^[^ ^13^, ^16^, ^17^, ^18^ ^]^ Thus, SERS sensors have not been used for astronaut health monitoring. Intriguingly, DNA origami nanostructures (DONs) are thought to hold promise for offering sequence‐specific binding sites that facilitate dependable single‐molecule detection by positioning the probe or target molecules precisely in origami column‐mediated hotspots.^[^ ^19^, ^20^, ^21^, ^22^, ^23^, ^24^, ^25^, ^26^, ^27^, ^28^, ^29^ ^]^ However, most DNA origami‐based plasmonic nanostructures remain proof‐of‐concept platforms that embed Raman dyes within the origami pillars. These structures typically lack responsiveness to specific chemical or biological stimuli. Additionally, most of them do not perform multiplexed or smart assays on clinical or preclinical samples.

To solve these dilemmas, we designed and constructed precision‐arranged DNA origami plasmonic nanoantennas for the first time. Using DNA origami, we precisely engineered antenna distributions on the microfluidic plasmonic substrate interface. This system, termed “u‐DON‐SERS”, integrates antenna arrays mediated by single‐layer DNA origami into silver nanoparticle‐modified silicon wafers (AgNPs/Si) to form a sandwich structure. By leveraging DNA origami's addressability, we constructed a 3 × 4 array with a controlled spacing of 21.76 nm (*d* × 4, *d* = 5.44 nm, i.e., the minimum lateral spacing of staple extension sites on DNA origami) and achieved an assembly yield of 77.2%. Moreover, the precision arrangement enhanced assembly efficiency fourfold compared to disordered systems (without DON, refer to “u‐SERS”). Utilizing this sensor, we simultaneously and accurately measured bone loss biomarkers of calcium ions (Ca^2+^), interleukin‐6 (IL‐6), and microRNA‐214 (miRNA‐214) in serum samples from mice subjected to 42 days of hind limb unloading and 1 Gray (Gy) radiation. Intraclass correlation coefficients (ICCs) > 0.75 confirmed the ordered system's reliable performance in biomarker detection, comparable to ELISA and qPCR. Furthermore, integrating the system with a convolutional neural network (CNN) enabled us to analyze serum samples from a 6° head‐down bed rest experiment for bone loss monitoring. The system achieved a high accuracy rate in predicting bone health status. We believe the proposed system will drive innovation in space biology and monitoring, with this work on bone loss serving as a proof of concept.

## 2 Results and Discussion

### 2.1 Design and Construction of u‐DON‐SERS Substrate

**Figure 1:**
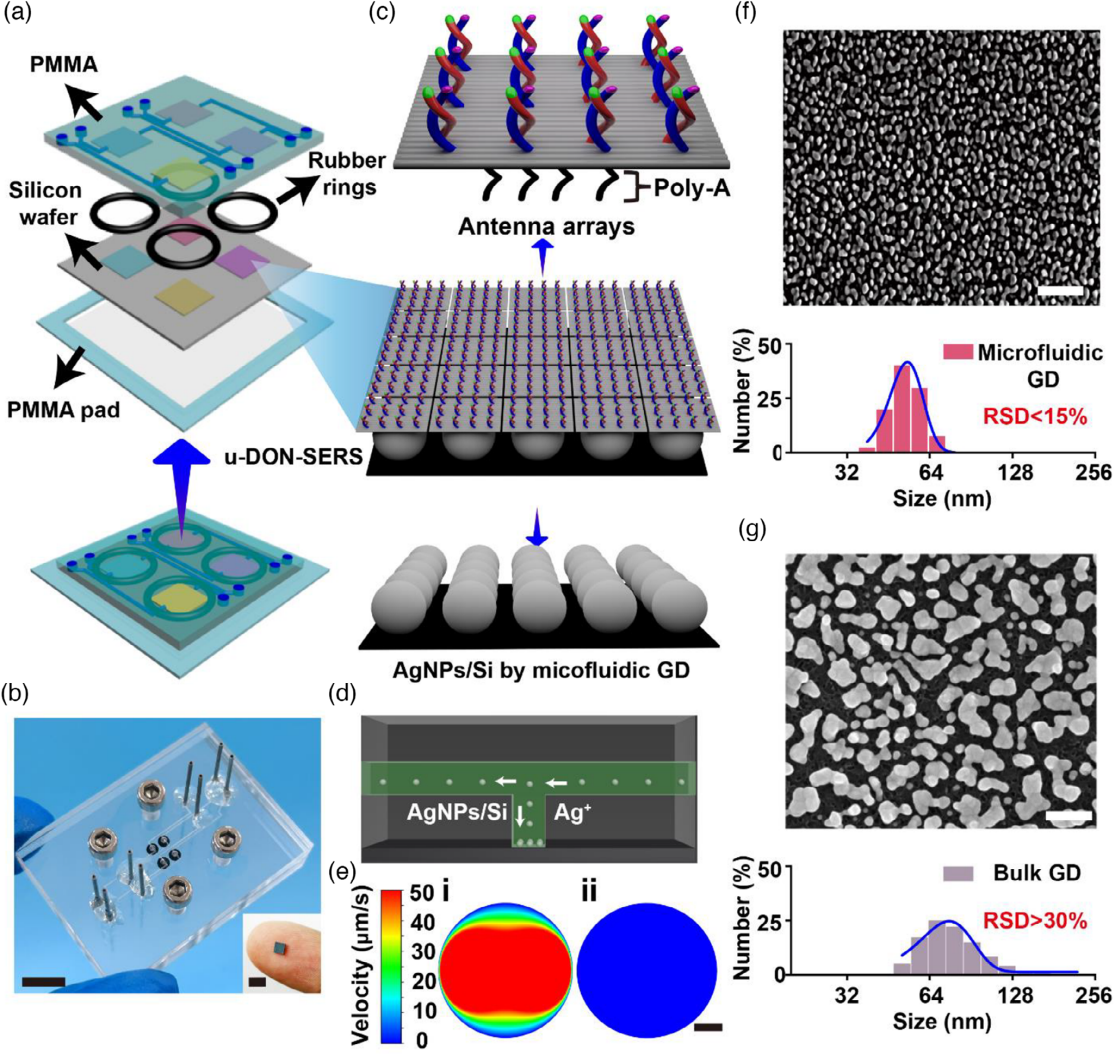
Construction of the u‐DON‐SERS substrate. a) Overview of the framework of the u‐DON‐SERS device. b) Actual picture of the microfluidic device and the silicon sensor, scale bar, 1 cm. c) Construction of u‐DON‐SERS. The lower layer consists of homogeneous AgNPs/Si prepared based on the microfluidic galvanic deposition (GD) reaction, and the upper layer consists of single‐layer DNA origami arrays. d) Formation of AgNP on the core surface in the microfluidic GD reaction. e) Cloud view of CFD simulated flow velocity field at 0.01 mm above the silicon in the microfluidic GD reaction (i) and the bulk GD reaction (ii). scale bar, 0.5 mm. f) Representative SEM images and DLS of AgNPs/Si synthesized via microfluidic GD reaction. scale bar, 100 nm. g) Representative SEM images and DLS of AgNPs/Si synthesized via Bulk GD reaction. scale bar, 100 nm. All imaging experiments were repeated three times with similar results.

The microfluidic device framework is shown in Figure **1**a and consists of three layers: the upper polymethylmethacrylate (PMMA) layer, the silicon wafer (0.5 × 0.5 cm^2^) in the middle layer, and the PMMA bottom pad in the lower layer. The PMMA substrate has four pairs of threaded ports (diameter: 5.4 mm), four microchannels (depth: 100 µm, width: 200 µm), and four isolated microcavities (height: 1 mm, diameter: 1 mm), and the flow paths are not interfering with each other, which allows for the simultaneous detection of multiple biomarkers. PMMA was chosen for its chemical resistance, thermal stability, light weight, transparency, and impact resistance.^[^ ^30^ ^]^ Prior to fabrication of the microfluidic reactor, the p‐silicon wafers were rinsed several times with distilled water and acetone and then ultrasonicated to remove impurities. To ensure the sealing performance of the constructed microfluidic reactor, four rubber rings (inner diameter: 1 mm, outer diameter: 1.5 mm); shown as black circles in Figure 1a) were sandwiched between the PMMA layer and the silicon layer. The microfluidic device and the silicon substrate are shown in Figure 1b.

Figure 1c illustrates the u‐DON‐SERS structure. The upper layer consists of single‐layer rectangular DNA origami array fabricated using the classic Rothemund method.^[^ ^31^ ^]^ The origami was synthesized by hybridizing M13mp18 (M13) single‐stranded DNA with shorter complementary strands, including polyadenine (poly‐A) containing segments that enable covalent assembly with silver nanoparticles.^[^ ^32^ ^]^ Additionally, site specific functional stretches were modified with cyanine 3 (Cy3) to enhance structural functionality (Figure S1, Tables S1 and S2, Supporting Information) (see Section 2 for details on precision engineering of plasmonic substrate interface). The assembly process involved gradual annealing from 65 to 25 °C, ultimately forming a 90 × 60 × 1 nm^3^ rectangular DNA origami.

To ensure the purity of the synthesized origami, a 100 kDa centrifugal filter was used to remove excess DNA. The successful formation of DNA origami was verified using multiple characterization techniques. Transmission electron microscopy (TEM) (Figure S2, Supporting Information) and atomic force microscopy (AFM) (Figure S3, Supporting Information) confirmed the expected 90 × 60 × 1 nm^3^ dimensions. Agarose gel electrophoresis (AGE) (Figure S4, Supporting Information) further validated the synthesis, as the DNA origami bands migrated significantly slower compared to M13 single‐stranded DNA, indicative of successful assembly. Finally, dynamic light scattering (DLS) measurements (Figure S5, Supporting Information) showed an average dynamic hydration diameter of ≈100 nm, closely matching the theoretical value of 90 nm. Collectively, these results confirm the successful synthesis of the DNA origami structure.

The lower layer comprised uniform AgNPs/Si prepared by microfluidic galvanic deposition (GD) reaction. In this process, silver nuclei form near the reactive silicon surface as Ag^+^ receives electrons from the silicon valence band (VB). Simultaneously, SiO_2_ forms beneath the AgNP and is etched away by HF solution. So once nucleation occurs, AgNP growth depends on the local Ag^+^ concentration (Figure 1d).^[^ ^33^, ^34^, ^35^ ^]^ To primarily investigate the flow velocity during the reaction, microfluidic transport was simulated using computational fluid dynamics (CFD) software (ANSYS Fluent). A single flow channel was precisely scaled down in a one‐to‐one manner and meshed using ANSYS Fluent Meshing, resulting in a final mesh count of 10^9^. To simulate the flow field under the microfluidic GD reaction and the bulk reaction, an initial flow rate of 1.35 mm s^−1^ was set for the former, while the latter was maintained at 0 mm s^−1^ (see Supporting Information for details). Figure S6 (Supporting Information) presents the flow profile of the entire flow channel under the microfluidic GD reaction. Additionally, the flow field distribution was visualized at a height of 0.01 mm above the silicon support, as shown in Figure 1e. The results indicated that, in the microfluidic GD reaction, the velocity field reached a maximum of 50 µm s^−1^ at this height. In contrast, in the bulk GD reaction, the velocity field at the substrate surface was nearly zero, meaning that ion transport occurs solely via free diffusion. This controlled convection in the microfluidic GD reaction significantly enhanced mass transfer, leading to a higher localized silver ion (Ag^+^) concentration near the reaction site. As a result, a more homogeneous and consistent growth of AgNPs was achieved. As expected, the microfluidic GD reaction minimized anisotropic Ag^+^ diffusion, producing AgNPs with a uniform size (53.05 ± 3.26 nm) and tight distribution (relative standard deviation (RSD) < 15%) (Figure 1f). In contrast, bulk GD reaction suffered from anisotropic ion diffusion, resulting in heterogeneous nucleation and variable growth rates (RSD > 30%) (Figure 1g). Therefore, the AgNPs/Si prepared via microfluidic GD exhibited more homogeneous distribution than those from bulk GD, ensuring uniform electromagnetic field enhancement (Figures S7 and S8, Supporting Information).

To confirm successful substrate integration with DNA origami, a DNA antenna array for IL‐6 detection was designed (see Section 2 for SERS substrate interface precision engineering). As shown in Figure S9 (Supporting Information), a Cy3‐modified capture strand (blue) was extended at a specific site on the DNA origami, while the IL‐6 aptamer was modified with rhodamine X (ROX). This ratio metric signal output strategy reduced nonspecific signal interference. Tables S3 and S4 (Supporting Information) listed the detailed modification sites and sequences. The successful u‐DON‐SERS assembly was verified by detecting the Raman peaks of Cy3 (1595 cm^−1^) and ROX (1507 cm^−1^) (Figure S10, Supporting Information).

### 2.2 Construction and Optimization of Precision‐Arranged DNA Origami Plasmonic Nanoantennas

**Figure 2:**
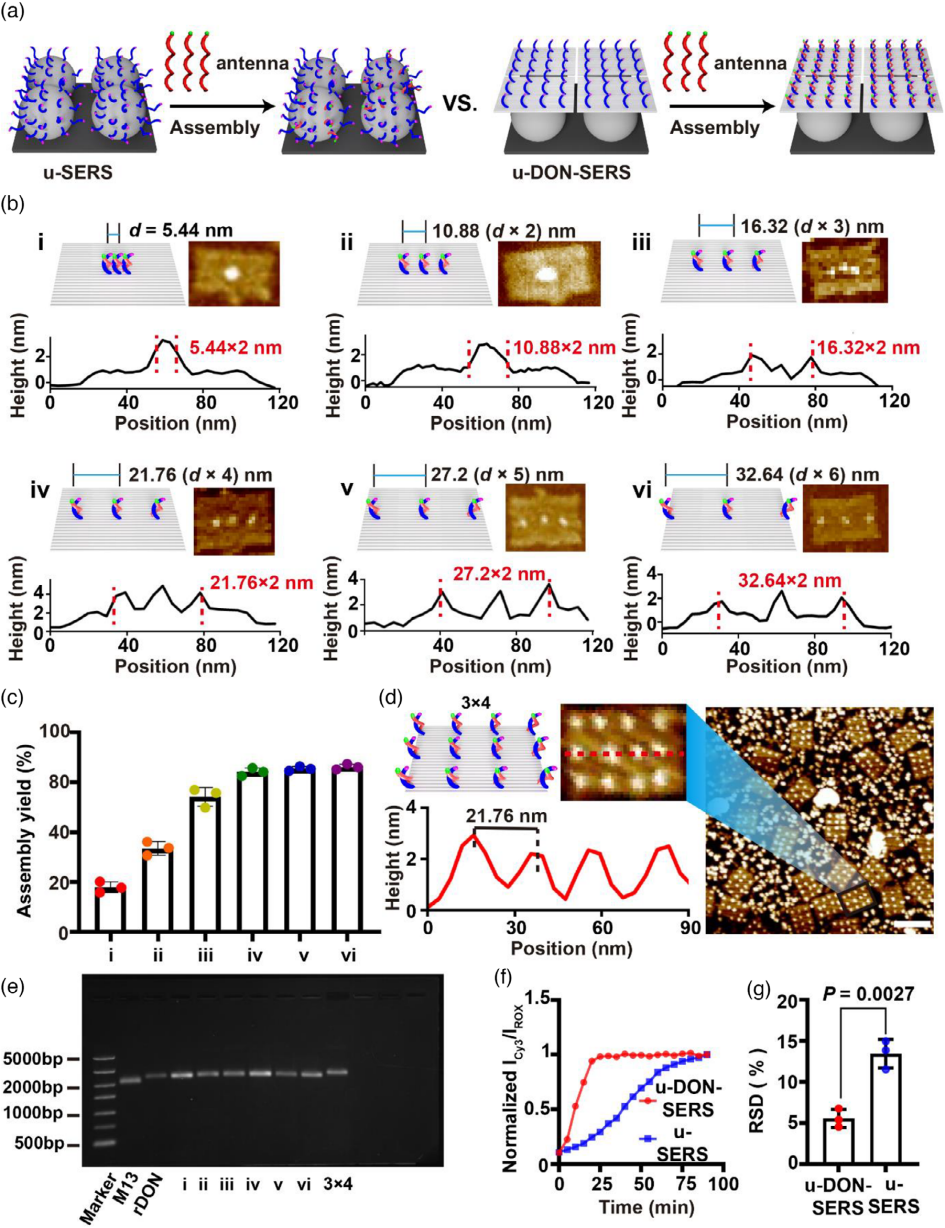
Construction and optimization of precision‐arranged DNA origami plasmonic nanoantennas. a) Schematic of u‐SERS and u‐DON‐SERS antenna assembly. b) (i‐vi) Representative AFM images and corresponding height profiles showing antenna spacings of 5.44 (*d* × 1) nm (i), 10.88 (*d* × 2) nm (ii), 16.32 (*d* × 3) nm (iii), 21.76 (*d* × 4) nm (iv), 27.2 (*d* × 5) nm (v), and 32.64 (*d* × 6) nm (vi). *d* = 5.44 nm, i.e., the minimum spacing of lateral staple extension sites on DNA origami. c) Assembly efficiency statistics for antenna spacings. d) AFM images and height profiles of the 3×4 antenna array per DNA origami (scale bar: 100 nm). e) AGE (1%) analysis of DNA origami loaded with different antenna spacings (bp: base pair). f) Assembly kinetics comparison between u‐DON‐SERS and u‐SERS. g) RSD comparison between u‐DON‐SERS and u‐SERS. Data are presented as mean ± standard deviation (SD). Error bars represent SD. *P* values in (g) were calculated using an unpaired two‐tailed t‐test (*P* < 0.05). Each imaging experiment was performed three times with consistent results.

The reaction interface critically influences the assembly efficiency, thermodynamic behaviour, and reproducibility of SERS sensors.^[^ ^36^ ^]^ Conventional homogeneous SERS platforms struggle with precision engineering of the reaction interface. Even in systems with well‐defined hotspot structures, probe assembly efficiency, kinetic performance, and signal reproducibility remain limited. In contrast, the u‐DON‐SERS platform exploits the nanoscale addressability of DNA origami to enable quasi‐homogeneous probe assembly on the substrate. This approach significantly enhances assembly kinetics and sensor reproducibility (Figure **2**a).

The classic Rothemund method produces rectangular DNA origami with a lateral staple extension site spacing of ≈5.44 nm (*d* = 5.44 nm).^[^ ^31^, ^37^ ^]^ Using this spacing as a fundamental unit, we systematically designed a series of 3 × 1 antenna array per rectangular DNA origami template with inter‐antenna distances of 5.44 (*d* × 1), 10.88 (*d* × 2), 16.32 (*d* × 3), 21.76 (*d* × 4), 27.2 (*d* × 5), and 32.64 (*d* × 6) nm (Figure 2b; Figure S11, Supporting Information). AFM imaging and corresponding height profiles (Figure 2b; Figure S12, Supporting Information) confirmed the successful fabrication of antennas with varying lateral distances. Assembly yield was quantified by counting fully assembled antennas per DNA origami (Figure 2c). antennas with 5.44 (*d* × 1) nm and 10.88 (*d* × 2) nm spacings exhibited low assembly yields of 22.2% and 42.1%, respectively, likely due to the relatively high steric hindrance. Increasing the spacing to 21.76 (*d* × 4) nm improved and stabilized the assembly yield at 80.3%. Beyond this distance, no significant gains were observed. Thus, we identified 21.76 (*d* × 4) nm as the optimal antennas spacing that maximizes efficiency while maintaining high antennas density.

Building on these findings, we designed a 3 × 4 antenna array per DNA origami with optimized antenna density. This array featured a lateral spacing of 21.76 (*d* × 4) nm and a longitudinal spacing of 24 nm (Figure S13, Supporting Information). AFM imaging and corresponding height profile analysis validated the successful formation of the 3 × 4 antenna array per DNA origami. Statistical analysis of large‐area AFM images showed a 77.2% antenna assembly yield, indicating high site occupancy, spatial uniformity, and efficient array fabrication (Figure 2d). AGE analysis (Figure 2e) revealed that the 3 × 4 antenna array per DNA origami exhibited a slower migration speed than all previously tested DNA origami, further confirming the successful preparation of 3 × 4 antenna array per DNA origami. To assess system stability in serum‐based biomarker detection, we incubated the u‐DON‐SERS sensor in 10% fetal bovine serum (FBS) for 24 h. AGE analysis confirmed the structural integrity of the rectangular DNA origami, indicating good stability (Figure S14, Supporting Information).

Next, we evaluated antenna‐substrate assembly dynamics in the u‐DON‐SERS system. To satisfy the core assumption of the pseudo‐first‐order kinetic model, an excess of aptamer was added to the sensor. Specifically, a 10 µL solution of 10 nM IL‐6 aptamer was added to both the ordered u‐DON‐SERS sensor and the disordered u‐SERS sensor. The intensity ratio of the ROX Raman peak (1507 cm^−1^) to the Cy3 Raman peak (1595 cm^−1^) was measured every 5 min for 90 min. Compared to the u‐SERS sensor, the u‐DON‐SERS system significantly enhanced assembly efficiency, reducing assembly time from 80 to 20 min (Figure 2f). We calculated the pseudo‐first‐order reaction rate (k′) using the following equation:
(1)

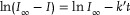

where, *I_∞_* was the final limit of the signal (after full reaction), *t* was the reaction time. The calculated value for the u‐DON‐SERS system (0.17 min^−1^) was ≈4 times higher than that of the u‐SERS system (0.04 min^−1^), demonstrating a significantly improved assembly rate. Furthermore, at 90 min, the RSD of the Raman signal ratio (I_ROX_/I_Cy3_) of the u‐DON‐SERS sensor (5.57%) was markedly lower than that of the u‐SERS sensor (13.45%) (Figure 2g). These enhancements resulted from the precise nanoscale control of the reaction interface enabled by DNA origami, a level of precision difficult to achieve with conventional probes. Consequently, we selected the 3 × 4 antenna array per DNA origami for subsequent experimental studies.

### 2.3 u‐DON‐SERS Sensor for Multiple Biomarker Analysis

Bone loss caused by microgravity and radiation poses a major challenge in manned space missions, primarily affecting weight‐bearing bones and prolonging recovery.^[^ ^2^, ^3^, ^4^, ^5^, ^38^, ^39^ ^]^ To address this, we developed precision‐arranged DNA origami plasmonic nanoantennas (u‐DON‐SERS) to serve as a liquid biopsy platform for detecting multiple bone loss biomarkers: Ca^2+^, miRNA‐214, and IL‐6. Specifically, Ca^2+^ reflects systemic calcium dysregulation associated with bone resorption under microgravity or disuse. Elevated serum calcium and urinary calcium excretion, accompanied by bone mineral loss, have been widely reported, making Ca^2^⁺ a reliable early indicator of bone resorptive activity.^[^ ^40^, ^41^ ^]^ IL‐6 is a pro‐inflammatory cytokine that promotes osteoclast differentiation and bone resorption. Its expression increases significantly under simulated microgravity, contributing to enhanced osteoclastic activity and reduced bone mineral density.^[^ ^42^, ^43^ ^]^ miRNA‐214 impairs bone formation by targeting ATF4, thereby suppressing osteoblast function and matrix mineralization. It is highly expressed in osteoclasts and can be transferred to osteoblasts, forming a negative feedback loop that inhibits bone formation. Its upregulation under microgravity has also been well documented.^[^ ^44^, ^45^ ^]^ The u‐DON‐SERS sensor, segmented into four functional zones, enables simultaneous detection of these biomarkers, improving diagnostic accuracy.

**Figure 3:**
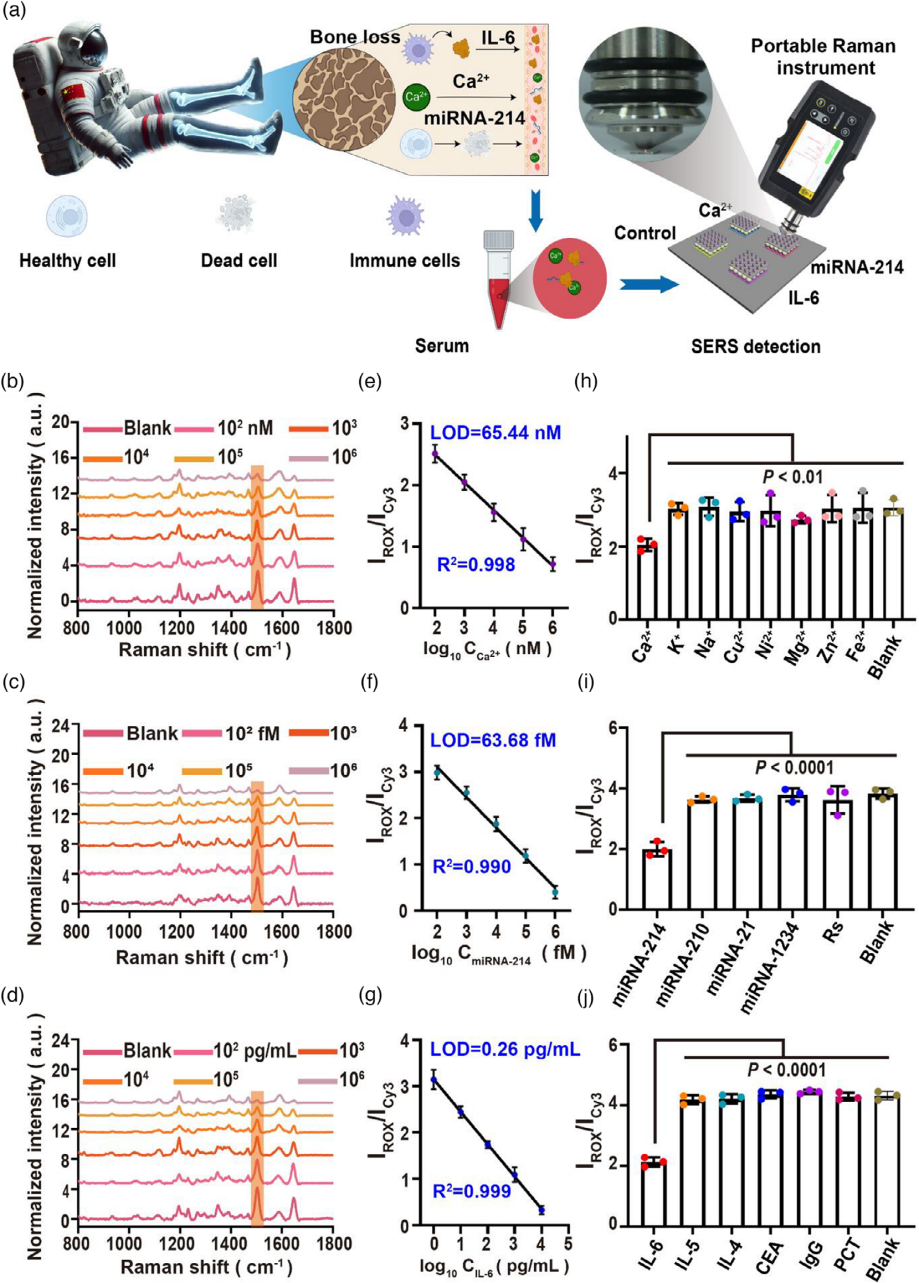
Multiplex biomarker detection using the u‐DON‐SERS sensor. a) Schematic of the u‐DON‐SERS sensor designed for detecting multiple bone loss biomarkers induced by microgravity and radiation. The image was created by BioRender (biorender.com). b‐d) Representative Raman spectra acquired from the u‐DON‐SERS sensor after exposure to increasing concentrations of Ca^2^⁺ (0 nM (blank), 100, 1, 10, 100, and 1 mM), miRNA‐214 (0 fM (blank), 100 fM, 1, 10, 100 pM, and 1 nM), and IL‐6 (0 pg mL^−1^ (blank), 1, 10, 100 pg mL^−1^, 1, and 10 ng mL^−1^) over 20 min. e‐g) Calibration curves quantifying Ca^2^⁺, miRNA‐214, and IL‐6 detection. h‐j) Analytical specificity of the u‐DON‐SERS sensor for ions (Ca^2^⁺, K⁺, Na⁺, Cu^2^⁺, Ni⁺, Mg^2^⁺, Zn^2^⁺, Fe^2^⁺ (1 µM each) and blank), nucleic acids (miRNA‐214, miRNA‐210, miRNA‐21, miRNA‐1234, random sequence RS (10 pM each) and blank), and proteins (IL‐6, IL‐5, IL‐4, CEA, IgG, PCT (100 pg/mL each) and blank). Raman signals were acquired at 785 nm excitation wavelength with medium power and a 1‐s integration time. Data are presented as means ± SD. Error bars = SD. Statistical significance in (h‐j) was determined using one‐way ANOVA with Dunnett's test.

For portable detection, we integrated the sensor with a compact Raman spectrometer (SERSTECH 100 Indicator, 785 nm excitation, 1 sec acquisition, medium laser power) (Figure **3**a). To ensure stability, both the spectrometer and sensor were secured on a fixed bracket (Figure S15, Supporting Information). Ultraviolet (UV) lithography was used to pattern distinct zones on a silicon wafer, including a control area containing nonfunctional DNA origami labelled with fluorophores Cy3 and ROX. Under standard conditions, the Raman signal ratio of Cy3 (1595 cm^−1^) to ROX (1507 cm^−1^) remained stable at 5.0, confirming consistent sensor functionality.

For Ca^2+^ detection, ROX‐labelled nucleic acid antennas served as detection signals, while complementary Cy3‐labelled strands acted as internal standards. Upon Ca^2+^ recognition, ROX‐labelled antennas dissociated, reducing the Raman signal (Figure S16, Supporting Information). For miRNA‐214 detection, a locked nucleic acid (LNA)‐mediated strand displacement reaction (LSDR) converted a Y‐shaped DNA structure into a hairpin upon miRNA‐214 binding, decreasing the ROX signal and increasing the Cy3 signal (Figure S17, Supporting Information). The ROX‐to‐Cy3 Raman signal ratio (I_1507_/I_1595_) enabled sensitive quantification of Ca^2+^ and miRNA‐214. Functional sequences and modification sites are detailed in Tables S5 and S6 (Supporting Information).

We then assessed the sensor's ability to detect these biomarkers. Within 20 min, the sensor successfully identified various concentrations of Ca^2+^, miRNA‐214, and IL‐6, generating SERS spectra with distinct signal intensities. The Cy3 peak at 1595 cm^−1^ served as a normalization reference. Without target biomarkers, a distinct Raman peak was observed. Upon target binding, the ROX signal at 1507 cm^−1^ weakened due to dissociation of the double‐stranded structure, releasing the ROX‐modified chain and reducing the signal (Figures 3b–d). The calibrated signal ratios showed strong linear correlations with target concentrations (Ca^2+^: correlation coefficient (R)^2^ = 0.998; miRNA‐214: R^2^ = 0.990; IL‐6: R^2^ = 0.999). The corresponding linear equations were Y = ‐0.4528×log10 (XCa^2^⁺) + 3.4, Y = ‐0.6543×log10 (XmiRNA‐214) + 4.4, and Y = ‐0.6983×log10 (XIL‐6) + 3.1, where Y was the normalized signal value (I_ROX_/I_Cy3_) and X was the target concentration (Figures 3e–g). The detection limits (LODs) were 65.44 nM for Ca^2+^, 63.68 fM for miRNA‐214, and 0.26 pg mL^−1^ for IL‐6 by setting the signal‐to‐noise ratio to 3:1. These values align with normal human serum concentrations (Ca^2+^: 2.03–2.54 mM; miRNA‐214: 1–2 pM; IL‐6: 1–5 pg mL^−1^),^[^ ^46^, ^47^, ^48^ ^]^ demonstrating the sensitivity of the developed sensor was sufficient for many clinical scenarios.

To evaluate reproducibility, we measured the Raman signals at 30 random points within a 500×500 µm^2^ detection area. The RSD of the Raman signal ratio (I_ROX_/I_Cy3_) was 5.79% for Ca^2+^ (Figure S18, Supporting Information), 5.05% for miRNA‐214 (Figure S19, Supporting Information), and 5.04% for IL‐6 (Figure S20, Supporting Information). This consistency resulted from the uniform plasmonic substrate and precise probe distribution. Next, we tested specificity by analyzing Raman signals under different conditions: ions (Ca^2+^, K^+^, Na^+^, Cu^2+^, Ni^2+^, Mg^2+^, Zn^2+^, and Fe^2+^(1 µM each) and blank), nucleic acids (miRNA‐214, miRNA‐210, miRNA‐21, miRNA‐1234, random sequences (RS) (10 pM each) and blank), and proteins (IL‐6, IL‐5, IL‐4, carcinoembryonic antigen (CEA), IgG, procalcitonin (PCT) (100 pg/mL each) and blank) (Figures 3h–j). Significant signal differences were observed (Ca^2+^: *P* < 0.01; miRNA‐214: *P* < 0.0001; IL‐6: *P* < 0.0001), confirming high specificity. In addition, the probe's specificity was further validated using both agarose gel electrophoresis (AGE) and a FRET‐based fluorescence assay. In the AGE analysis, DNA origami nanoantennas were incubated with target analytes (Ca^2^⁺, miRNA‐214, and IL‐6) or non‐target controls (other ions, miRNAs, and proteins) in a serum‐mimicking buffer. Upon specific recognition, the aptamer strand was released or displaced, resulting in a reduced molecular weight and a downward shift in gel mobility (Figures S21–S23, Supporting Information). In contrast, non‐target molecules induced no shift, and band patterns remained identical to the blank control, indicating negligible interaction. To further validate target specificity, we implemented a FRET‐based detection scheme by labeling the origami's extension strand with FAM dye and the aptamer strand with BHQ1. Target binding triggered aptamer dissociation, disrupting FRET quenching and restoring fluorescence. Significant fluorescence recovery was observed exclusively with IL‐6, miRNA‐214, or Ca^2^⁺ (Figures S24–S26, Supporting Information), while non‐targets produced minimal signal change. Together, these results confirm the high molecular specificity of the aptamer‐based recognition system, even under biologically relevant conditions.

To evaluate the reproducibility of sensor fabrication, SERS signals were measured across five independent batches of silicon‐based SERS substrates. For each batch, we measured SERS signals at five random spots for Ca^2^⁺, miRNA 214, and IL 6 detection. The relative standard deviations (RSDs) were 7.1%, 8.8%, and 10.5%, respectively (Figures S27–S29, Supporting Information). These low RSDs demonstrate robust manufacturing consistency, which is vital for large scale production and space mission applications. More interestingly, the highly ordered structure and optimal probe arrangement enhanced recognition efficiency, reducing detection time to 20 min‐significantly faster than conventional SERS sensors, which required over an hour.^[^ ^49^, ^50^ ^]^

### 2.4 Preliminary Validation of u‐DON‐SERS Sensor Using the Mouse Hindlimb Unloading Model

**Figure 4:**
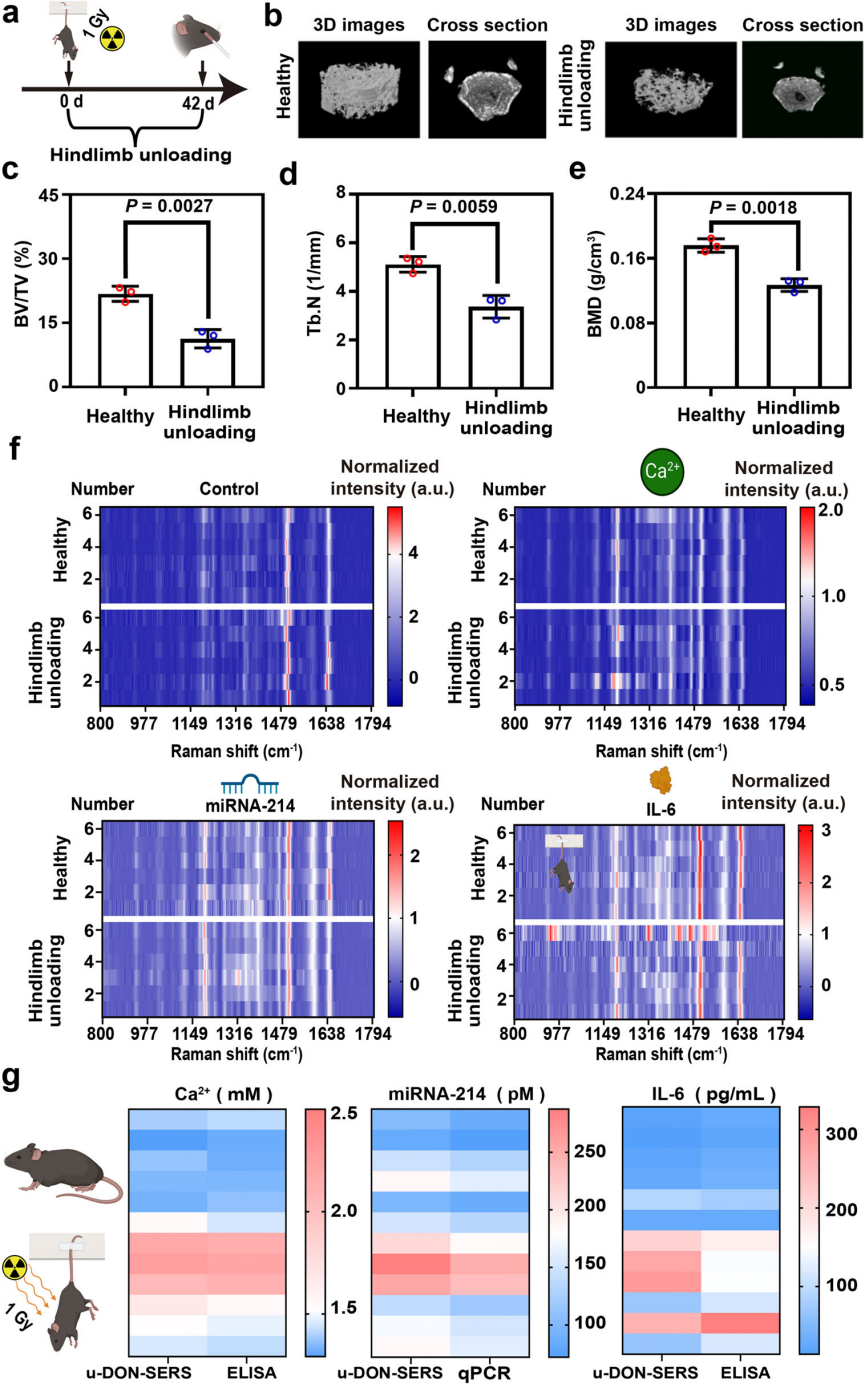
Performance evaluation of the u‐DON‐SERS sensor in a bone loss model. a) Illustration of the experimental design depicting the mouse model for bone loss. b) 3D micro‐CT reconstructions of mouse support bones. c‐e) Quantitative analysis of BV/TV, BMD, and Tb.N in control and hindlimb‐unloaded mice. f) Raman spectra of serum samples from control mice (n = 6) and irradiated mice subjected to 42 days of hindlimb unloading (n = 6). g) Double‐blinded quantification of Ca^2^⁺, miRNA‐214, and IL‐6 levels in serum using the u‐DON‐SERS sensor, cross‐validated against ELISA and qPCR. Data are presented as means ± SDs, with error bars representing SDs. P values in (c), (d), and (e) were determined using an unpaired two‐tailed t‐test. Each imaging experiment was performed in triplicate, yielding consistent results.

The mouse hindlimb unloading (Hu) model simulates bone loss under microgravity.^[^ ^44^ ^]^ To mimic spaceflight‐induced bone deterioration, C57BL/6 mice (6‐8 weeks, ≈20 g) received 1 Gy of radiation and underwent 42 days of hindlimb unloading (Figure **4**a). Micro‐CT analysis assessed bone microstructure by measuring key parameters, including bone volume/total volume (BV/TV), bone mineral density (BMD), trabecular number (Tb.N), trabecular thickness (Tb.Th), trabecular separation (Tb.Sp), and the structural model index (SMI). Compared with controls, the irradiated and tail‐suspended groups exhibited substantial bone mass loss (Figure 4b; Figure S30, Supporting Information). Specifically, BV/TV and BMD decreased by 48.1% and 29.4%, respectively, while Tb.N and Tb.Th declined by 34.1% and 21.6%. Conversely, Tb.Sp and SMI increased by 17.2% and 11.9% (Figures 4c–e; Figures S31a–c, Supporting Information). These findings validated the robustness of the Hu model for studying bone health under microgravity and radiation.

Serum samples from control and irradiated mice were analyzed using the u‐DON‐SERS sensor. A blinded analysis visualized the results as a heatmap (Figure 4f). In the control group, the ratio of the Cy3 Raman peak (1595 cm^−1^) to the ROX Raman peak (1507 cm^−1^) remained stable at ≈5.0, confirming sensor consistency. The concentrations of Ca^2^⁺, miRNA‐214, and IL‐6 were quantified using linear regression equations and cross‐validated against ELISA and qPCR (Figure 4g). We used the ICCs to determine consistency between the methodologies. The ICCs formula was as follows:

(2) $$ICCs=\frac{\sigma_{T}^{2}}{\sigma_{T}^{2}+\sigma_{B}^{2}+\sigma_{E}^{2}}$$

where $\sigma_{T}^{2}\;$ denoted the variance due to the measured entity, $\sigma_{B}^{2}\;$ represented the systematic error, and $\sigma_{E}^{2}$ accounted for the random error. The ICCs values ranged from 0 to 1, with values below 0.5 indicating poor agreement, 0.5‐0.75 indicating moderate agreement, 0.75‐0.9 indicating good agreement, and values above 0.9 indicating excellent agreement. ICCs analysis showed good consistency between SERS and ELISA for IL‐6 (ICCs > 0.75) and excellent consistency between SERS and qPCR for Ca^2^⁺ and miRNA‐214 (ICCs > 0.9). These results confirmed the reliability of the u‐DON‐SERS sensor in biomarker detection, comparable to ELISA and qPCR. To assess its practical utility, we compared the u‐DON‐SERS platform with ELISA, qPCR, and mass spectrometry (MS) across six operational criteria, including assay time, sample pretreatment, equipment complexity, and field applicability (Table S7, Supporting Information). ELISA and qPCR require 2.5–4 h and trained personnel due to multistep workflows and sensitive reagents. MS, though highly sensitive, relies on bulky instrumentation. By contrast, u‐DON‐SERS yields results within ≈20 min, requires minimal pretreatment, and operates with simple, portable equipment. Its DNA origami interface ensures robust performance under extreme conditions, making it well‐suited for point‐of‐care diagnostics in resource‐limited or spaceflight environments.

### 2.5 Deep Learning‐Assisted u‐DON‐SERS Sensor for Early Warning of Human Bone Loss

To enable real‐time and intelligent detection of astronaut bone loss in extreme space environments, we developed a deep learning‐assisted u‐DON‐SERS sensing platform for bone health assessment. This system integrates a u‐DON‐SERS sensor, a SERS spectral processing module, and an intelligent analysis unit, providing a streamlined approach for early diagnosis. The SERS spectral processing module applies baseline correction, noise reduction, and signal normalization to spectra collected from the u‐DON‐SERS sensor, generating quantitative outputs (Figure S32, Figure S33, Supporting Information). The deep learning module within the intelligent analysis unit detects early signs of bone health deterioration and issues timely alerts.

**Figure 5:**
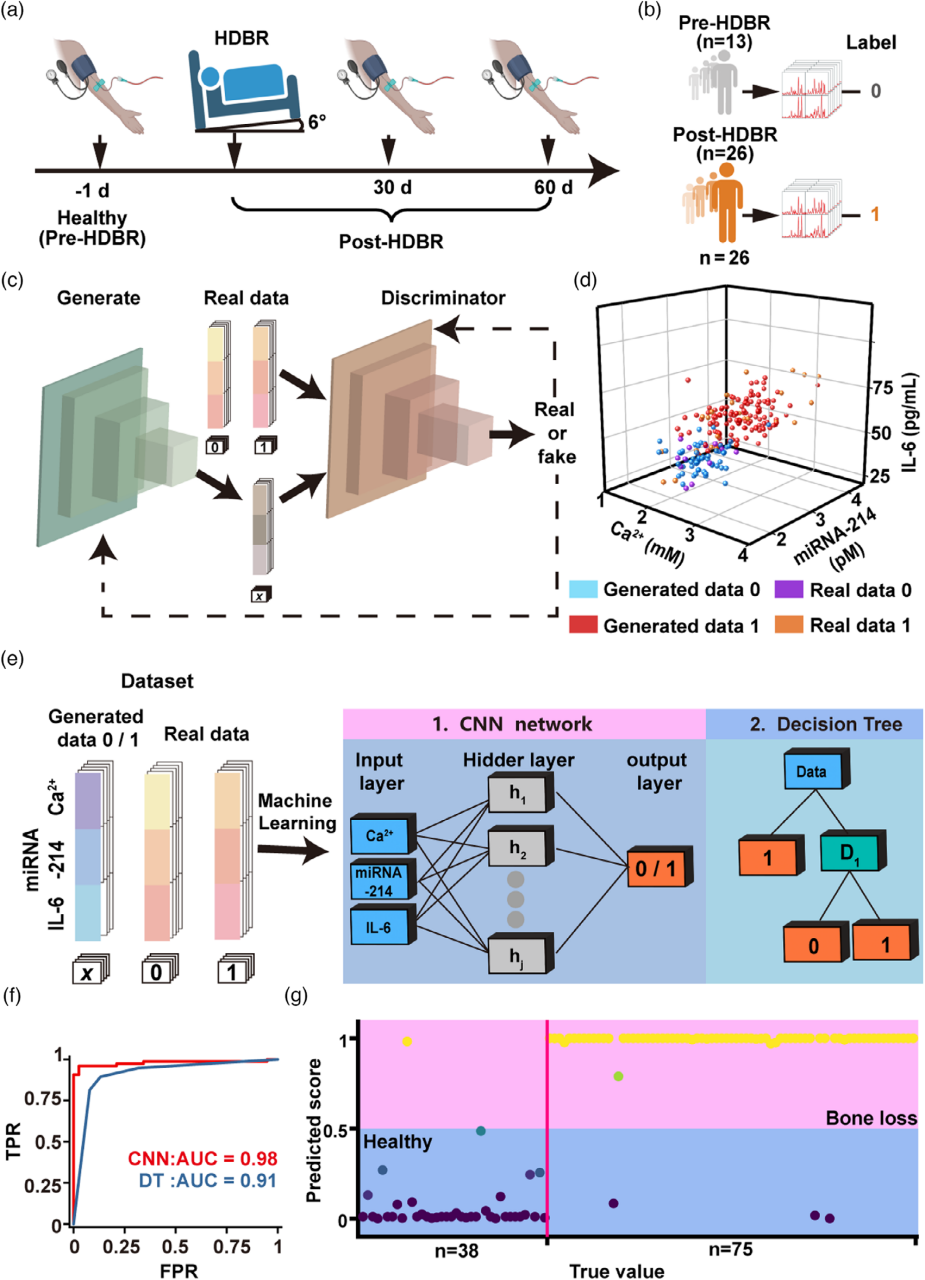
Deep learning‐assisted u‐DON‐SERS sensor for monitoring and early warning of bone loss. a) Schematic representation of the HDBR protocol simulating microgravity, with blood samples collected before (pre‐HDBR, day –1) and after HDBR (Post‐HDBR, day 30 or 60). The image was created by BioRender (biorender.com). b) Samples were labelled based on bone loss status: healthy (n = 13, label = 0) and bone loss (n = 26, label = 1). The image was created by BioRender (biorender.com). c) Synthetic data generation using a GAN, where the generator learns to produce realistic data indistinguishable from real samples by the discriminator. d) 3D visualization of the distribution of real and generated data in the biomarker space defined by Ca^2^⁺, miRNA‐214, and IL‐6 levels. e) Combined dataset (real + generated) used for machine learning. Two models were trained: CNN and DT, using Ca^2^⁺, miRNA‐214, and IL‐6 as input features. f) Receiver operating characteristic (ROC) curves and area under the curve (AUC) values for CNN and DT models on the test set. g) CNN predicted scores of all testsamples. True value means test data generated by GAN model.

We validated the system using a 6° head‐down bed rest (HDBR) study, a well‐established model for simulating microgravity‐induced physiological changes and evaluating countermeasures.^[^ ^51^, ^52^ ^]^ Due to the number of volunteers, sampling frequency, and ethical limitations, we only collected serum samples from participants at three key time points: baseline (before HDBR), day 30, and day 60 (Figure **5**a).

The u‐DON‐SERS system analyzed key biomarkers, including Ca^2^⁺, miRNA‐214, and IL‐6. As previously reported, prolonged exposure to head‐down bed rest (HDBR) simulating microgravity was associated with a gradual decline in bone mass.^[^ ^52^ ^]^ We classified baseline serum samples as the “normal group” (label = 0) and samples from days 30 and 60 as the “abnormal group” (label = 1) (Figure 5b).

A limited sample size often constrained deep learning performance due to insufficient training data. To address this, we incorporated a generative adversarial network (GAN) to augment the dataset. GANs generated high‐fidelity synthetic data and were widely used in image synthesis, medical data augmentation, and natural language processing.^[^ ^53^, ^54^ ^]^ The GAN model employed adversarial training, where a generator produced synthetic data resembling real samples, while a discriminator learned to distinguish between real and synthetic instances (Figure 5c). Iterative training refined the generator, enhancing data fidelity. Using this approach, we generated 100 synthetic samples for the normal group and 200 for the abnormal group. Validation confirmed that the synthetic data closely matched the distribution of real samples (Figure 5d), demonstrating the effectiveness of GAN‐driven dataset expansion.

To optimize classification accuracy, we combined real and synthetic samples to form a comprehensive dataset. We randomly split the dataset into a training set (226 samples) and a test set (113 samples) in a 2:1 ratio. To improve generalizability and mitigate overfitting, we employed tenfold cross‐validation during training. This ensured each subset contributed to model validation, reducing performance fluctuations due to sampling bias.^[^ ^55^, ^56^ ^]^ We then evaluated two machine learning architectures: a convolutional neural network (CNN) and a decision tree (DT) (Figure 5e). Both models demonstrated strong performance, with CNN achieving the area under the curve (AUC) of 0.98 and DT the AUC of 0.91 on the test set (Figure 5f). CNN's superior pattern recognition ability underscored its suitability for detecting complex biological signals. To further assess model performance, we visualized the predicted scores from both classifiers (Figure 5g; Figure S34, Supporting Information) and provided the corresponding confusion matrices for the CNN and DT models (Figures S35 and S36, Supporting Information). Plotting the true labels (healthy or bone loss) against predicted scores revealed CNN's superior discriminative capability, producing more distinct and consistent class separation. Quantitative analysis confirmed these findings: CNN achieved an accuracy of 96.50%, sensitivity of 96.00%, and specificity of 97.36%, outperforming DT (88.50%, 89.30%, and 86.84%, respectively). Therefore, we consider the CNN model as the preferred model for investigation. These results establish CNN as the preferred model for this application. To rigorously evaluate the reliability of the CNN model, we conducted a comprehensive assessment of its convergence behavior, predictive performance, and generalization capacity. As shown in the learning curves (Figure S37, Supporting Information), both training and validation losses decreased sharply during the initial epochs and plateaued by the fifth epoch. The final loss difference remained below 0.02, indicating smooth convergence with no evidence of overfitting. In addition, to further assess classification performance, we included a comprehensive report (Table S8, Supporting Information) detailing precision, recall, F1‐score, and accuracy for each class. These metrics confirm that the model achieves balanced and robust performance, even in the presence of potential class imbalance. Collectively, these findings demonstrate that the CNN model exhibits stable convergence, minimal overfitting risk, and reliable predictive performance across data partitions, supporting its promise as a viable tool for early bone loss detection in astronaut health monitoring applications.

## 3 Conclusion

SERS holds significant potential for astronaut health monitoring, particularly for assessing microgravity and radiation‐induced physiological changes. Conventional homogeneous SERS platforms lack precisely engineered reaction interfaces, limiting probe assembly efficiency and thermodynamic control. Here, we addressed this challenge by integrating DNA origami (≈90 × 60 × 1 nm^3^) onto a SERS sensor (≈1 mm^2^) composed of AgNPs/Si. This architecture optimized probe interspacing to 21.76 nm, achieving a 77.2% assembly yield and configuring probe arrays in a 3 × 4 arrangement. This engineered interface significantly improved probe assembly efficiency and thermodynamics. The pseudo‐first‐order reaction rate constant (k′) increased from 0.04 min^−1^ on an unmodified sensor to 0.17 min^−1^ on the engineered platform. The optimized sensor precisely quantified Ca^2^⁺, IL‐6, and miRNA‐214 in serum samples from mice subjected to 42 days of hind limb unloading and 1 Gy radiation. Intraclass correlation coefficients exceeding 0.75 validated biomarker reliability, matching ELISA and qPCR standards. Additionally, CNN integration enabled accurate bone health prediction. Although the present study was conducted under terrestrial laboratory conditions, we acknowledge the importance of advancing SERS‐based platforms for spaceflight applications.

Notably, China's Tiangong Space Station is already equipped with a Raman spectrometer for onboard material and biological analyses, providing a technical foundation for in‐orbit implementation of SERS diagnostics (China Space Station Scientific Experiment Resource Manual, 2019). Given the intrinsic advantages of SERS—such as compactness, low power consumption, label‐free detection, and high sensitivity—its integration into space biomedical payloads is both technically feasible and mission‐relevant. Moreover, recent advances in microfluidics and lab‐on‐a‐chip technologies have demonstrated reliable fluid handling and reaction performance under microgravity. These developments support automated sample processing and biomarker detection in sealed, miniaturized formats ideally suited for operation in vibration‐sensitive, confined environments like spacecraft. Furthermore, given the popularity of space travel, using volunteers of different ages, genders, and ethnicities for HDBR experiments is expected to improve generalizability and clinical translation. This study was conducted as a proof of concept, and all participants were young and healthy males to minimize biological variation during the proof‐of‐concept phase. We intentionally selected this homogeneous cohort to isolate the effects of simulated microgravity and reduce confounding factors from age‐ or gender‐related hormonal effects. Future studies will include larger, demographically diverse cohorts. Overall, the u‐DON‐SERS platform provides a robust, modular solution for advanced bioanalysis and in‐orbit health surveillance and holds strong potential for future deployment in long‐duration space missions.

## 4 Experimental Section

### Materials and Instrumentation

M13mp18 single‐strand DNA was obtained from BIORULER. DNA oligonucleotides were synthesized, modified, and purified by Sangon Biotech (Shanghai, China). GelRed, agarose, and reagents for buffers were obtained from Sangon Biotech. TAE/Mg^2+^ buffer (1X) was acquired from Biotech. Standard ion solutions such as Na^+^ and Fe^2+^ were purchased from Aladdin (Shanghai, China). Protein standards (IL‐6 and IL‐5) were obtained from Sangon Biotech. ELISA kits were obtained from Youpin Biotech (Wuhan, China). Serum miRNA extraction kits were purchased from Suzhou Ruijing Biotechnology, and miRNA reverse transcription kits were purchased from Kangwei Biotech (Taizhou, China). The primers used for the qPCR of miRNA‐214 were from Ruibo Biotech (Guangzhou, China), and the PCR detection kit was from Novezan (Nanjing, China).

### Microfluidic Device

The microfluidic device has three layers: an upper PMMA microfluidic sensor with 4 pairs of threaded ports (5.4 mm diameter), 4 microchannels (100 µm depth, 200 µm width), and 4 through‐hole cylindrical microcavities (1 mm height, 1 mm diameter). The middle layer is a p‐type silicon wafer, cleaned and ultrasonically treated, from Hefei Kejing Science and Technology. Four fluororubber sealing rings (1 mm inner diameter, 1.5 mm outer diameter) ensure effective sealing between the PMMA and silicon layers. The base layer is a PMMA sensor. The device was processed by Suzhou Wenhao Microfluidic Technology Co., Ltd.

### Preparation of the Silver Nanoparticle‐Modified Silicon‐Based SERS Substrate

A cleaned silicon wafer (50 mm × 50 mm) was integrated with a PMMA sensor to construct a microfluidic reactor. Initially, a 5% hydrofluoric acid (HF) solution was introduced at a flow rate of 6 µL min^−1^ for 25 min to remove the native silicon dioxide layer and generate hydrogen‐terminated silicon surfaces. Subsequently, a 10% HF solution containing 2.5 mM silver nitrate (AgNO_3_) was introduced at the same flow rate for 12 min to facilitate the in situ deposition of silver nanoparticles (Ag NPs). The reaction was terminated by flushing with deionized water, yielding a uniform Ag NPs@Si SERS substrate. After preparation, the substrates were immediately dried under a gentle nitrogen stream and stored in sealed containers under a nitrogen atmosphere to prevent oxidation of the Ag NPs and preserve their SERS activity. Substrate morphology and nanoparticle distribution were characterized by scanning electron microscopy (SEM, SU8230) and Zeta potential analysis (Nano ZS90), confirming the formation of a uniform and stable SERS‐active surface.

### Computational Fluid Dynamics Simulations

Computational fluid dynamics (CFD) simulations were conducted via Ansys Academic Research Fluent (Release 2020 R2) with a double‐precision steady‐state solver. The geometry of the microchambers in the microfluidic sensor was designed in Ansys SpaceClaim at a 1:1 scale to reflect the actual size of the experimental setup. The geometry was then meshed via the watertight geometry workflow of Ansys Fluent Meshing, resulting in a grid of 10^9^ polyhedral cells, ensuring accurate representation of fluid flow within the microchambers. The fluid flow was calculated via the laminar flow model. The output flow rate was 1.35 mm/s. The output parameter was the pressure outlet. The solver scheme was coupled. The simulation was run until the residuals for continuity, x‐, y‐, and z‐ velocities, turbulent kinetic energy (k), and kinetic energy dissipation rate (e) reached 10^−5^.

The flow within the microchambers was assumed to be laminar owing to the low Reynolds number typically observed in microfluidic systems. The Navier‒Stokes equations governing the conservation of momentum and the continuity equation for incompressible, steady‐state laminar flow were employed:

Governing Equations:

The Continuity Equation (Mass Conservation):

(3) $$\nabla \cdot \vec{v}\;=0$$

where $\vec{v}$ denotes the velocity vector field.

Navier‒Stokes Equations (Momentum Conservation):

(4) $$\rho \left(\vec{v}\cdot \nabla \vec{v}\right)\;=-\nabla p+\mu \nabla^{2}\vec{v}$$

where ρ indicates the fluid density (*kg/m^3^*), μ represents the dynamic viscosity (*Pa·s*), and p represents the pressure (*Pa*).

These equations were solved iteratively via the finite volume method implemented in Ansys Fluent.

### Finite‐Difference Time‐Domain (FDTD) Simulation

To investigate the electromagnetic field enhancement of uniformly distributed silver nanoparticle arrays, FDTD simulation was performed via FDTD Solutions software (Lumerical Co. Ltd.). In this study, plane waves were employed as the source of illumination. Periodic boundary conditions were applied in the X‐ and Y‐axes, whereas perfectly matched layer (PML) boundaries were applied along the Z‐axis. The mesh size was set to 0.5 nm.

### Raman Spectroscopy Measurements

Raman spectra were acquired using a portable Raman spectrometer (SERSTECH, Serstech 100 Indicator), with the sensor and instrument fixed on a custom bracket to maintain a stable working distance. Excitation was carried out using a 785 nm laser at medium power, with an integration time of 1 s. Prior to measurement, the Raman system was calibrated using the built‐in polystyrene standard embedded in the protective cap of the Raman probe to ensure spectral accuracy. All spectral data were processed using Origin software.

### Preparation of DNA Origami Template

DNA origami was prepared by mixing long M13mp18 single‐strand DNA with short and functional chains in 1× TAE‐Mg^2+^ buffer (40 mM Tris, 20 mM acetic acid, pH 8.0; 2 mM EDTA; 12.5 mM Mg^2+^). The ratio of scaffold DNA to short chains was 1:8. The mixture was heated to 65 °C and annealed at 25 °C for a period of 3 h via an ABI 96‐well gradient PCR instrument. The excess short chains were subsequently removed via an Amicon Ultra 0.5 mL 100 kDa centrifugal filter (Millipore). The DNA origami was precipitated with 200 µL of 1× TAE‐Mg^2+^ buffer, subjected to centrifugation at 3,000 × g for 10 min, washed twice with buffer, and centrifuged at 3,000 × g for 10 min each. The remaining solution was collected by centrifugation at 1,000 × g for a period of 10 min. The origami was characterized via atomic force microscopy (AFM) (Bruker).

### Hindlimb Unloading Mouse Model

The C57BL/6 mice, aged 6‐8 weeks, were maintained under specific laboratory conditions, which included a temperature of 25 °C, relative humidity of 55‐60%, and a 12‐h light/dark cycle. All procedures were approved by the Soochow University Laboratory Animal Center (approval number: SUDA2030619A04). Tail suspension was employed as a means of simulating weightlessness‐induced bone loss, thereby eliminating the mechanical load on the hindlimbs. The mice were housed individually, with their tails suspended at an angle of 30° to the floor. They were permitted to move freely and access food and water. Following a 42‐day period, blood samples were collected, and bilateral femurs and tibias were dissected for micro‐CT examination.

### Micro‐CT Analysis

The bone phenotype of the mice was analysed via a micro‐CT system (Scanco Medical, μ‐40, Switzerland). Each distal femur was scanned with 634 slices at a voxel size of 10.5 µm, with 80 consecutive slices of interest selected from the growth plate to the distal end of the femur (210 µm trabecular bone). For the purpose of measuring the cortical region, 80 consecutive sections were selected in the diaphyseal region, commencing at a distance of 3.57 mm from the growth plate. The parameters BMD, BV/TV, Tb.N, Tb.Th, Tb.Sp, and Cort.Th were calculated via 3D reconstruction of the sections.

### qPCR Detection of Serum miRNA‐214

Serum microRNA was extracted via a rapid extraction kit (Aidlab) and reverse transcribed to obtain cDNA via a reverse transcription kit (HiFiScript gDNA Removal RT MasterMix). The conditions for cDNA synthesis were as follows: incubation at 37 °C for 15 min and 85 °C for 5 s. A volume of 8.4 µL of cDNA, 0.8 µL of miRNA‐214 forward and reverse primers (Raybo Bio), and 10 µL of 2×Taq Pro Universal SYBR qPCR Master Mix (Novizan) were subsequently added to the qPCR instrument (ViiA 7). The qPCR program was as follows: Stage 1 (predenaturation) 95 °C, 30 s; Stage 2 (cyclic reaction) 95 °C, 3‐10 s, 60 °C, 10‐30 cycles (40 cycles); and Stage 3 (melting curve) utilizing the instrument's default program.

### Head‐Down Bed Rest Experiment

A total of 13 healthy male subjects of Asian ethnicity (mean ± SEM: age 30 ± 1 years, weight 62 ± 1 kg, height 169 ± 1 cm) participated in the Earth Star International Bed Rest Experiment. All participants were free of major illnesses and had no history of recent antibiotic use. Prior to enrolment, the volunteers were informed of the study's procedures and potential risks associated with the study, and written informed consent was obtained. The experimental procedures were approved by Ethics Committee (Reference no. CHEC2023‐168). The experiment was conducted over a period of 90 days and comprised of three distinct phases: a 15‐day pre‐HDBR adaptation period (B‐15 to B‐1), 60 days of head‐down bed rest (HDBR; D1 to D60), and a 15‐day recovery phase post‐HDBR (R+1 to R+15). During the course of the study, the participants were required to adhere to strict dietary and lifestyle protocols. Nicotine, alcohol, tea, and caffeine consumption were prohibited. A controlled diet providing 2400–2900 kcal/day was administered, while water intake remained unrestricted. To ensure consistency, the participants adhered to a fixed sleep schedule, woke at 6:30 am, and retired at 10:30 pm, with ambient room temperatures maintained at 23–25 °C. Serum samples were collected and monitored at three pivotal time points throughout the course of the study.

### Generative Adversarial Network Algorithm

All data were subjected to preprocessing via bespoke Python scripts. The features were standardized via StandardScaler, and labels were retained for subsequent integration. A generative adversarial network was constructed via TensorFlow 2.x and the Keras API. The generator model consisted of fully connected layers with batch normalization, Gaussian noise, and leaky ReLU activations, with the objective of increasing variability and robustness. The discriminator model uses dense layers with ReLU activations and a sigmoid output for binary classification between real and generated data. The training of the GAN was conducted for 500 epochs using a batch size of 16. To enhance the stability of the training process, an adaptive noise variance strategy was employed, with a range of 0.1‐1.0. To guarantee diversity, a minimum difference threshold was imposed between the generated samples. Synthetic samples were generated, standardized, and subsequently inverse‐transformed to match the original data distribution. The generated data were integrated with the original dataset, including the label column, to create an augmented dataset. The combined dataset was exported as a CSV file, thus providing an enriched data resource for further analysis or model training.

### CNN Network Algorithm

All the data were processed via custom Python scripts. A CNN was implemented via TensorFlow 2.x and the Keras API. The network architecture incorporated convolutional Conv1D layers with ReLU activation for feature extraction, dropout for regularization, and fully connected layers with Softmax activation for binary classification. The dataset, comprising features “Ca^2+^”, “miRNA‐214”, and “IL‐6”, was normalized, and labels were encoded via the one‐hot method. The data were divided into a training set (two‐thirds of the total) and a test set (one‐third of the total) via a stratified sampling method. A 10‐fold stratified cross‐validation was conducted, with the resulting AUC‒ROC and precision‒recall curves calculated for each fold. The learning rate, batch size, and number of epochs were optimized manually. An AUC score was obtained from the evaluation of the independent test set, and the performance metrics and visualizations were saved for further analysis.

### Statistical Analysis

All the experiments were performed independently at least three times. The data are presented as the means ± SDs. A two‐tailed t test was used for comparisons between two groups, whereas one‐way analysis of variance (ANOVA) with Dunnett's test was used for multiple group comparisons via GraphPad software. *P* values <0.05 were considered statistically significant.

## Conflict of Interest

The authors declare no conflict of interest.

## Supporting information



Supporting Information

## Data Availability

The data that support the findings of this study are available from the corresponding author upon reasonable request.
