# Supplementary material for: Precision‐Arranged DNA Origami Plasmonic Nanoantennas for Multidimensional Smart‐Warning of Weightlessness Induced Bone Loss
Source: Adv Sci (Weinh). 2025 Aug 11;12(40):e07189. doi: 10.1002/advs.202507189 (PMC12561424; doi:10.1002/advs.202507189)
Supplement: Supplementary file 1 — Supporting Information [file ADVS-12-e07189-s001.docx]

**Supporting Information**

**Precision-Arranged DNA Origami Plasmonic Nanoantennas for Multidimensional Smart-Warning of Weightlessness Induced Bone Loss**

*Yufan Ling,^1^ Xuan Qin,^2^ Weijia Sun,^3^ Fan Yue,^2^ Yiwei Wang,^1^ Di Fan,^2^ Haoyuan Xu,^2^ Ruao Xie,^2^ Jiawei Zhang,^2^ Jianwei Li,^3^ Bingyan Li,^4^ Yang Yang,^5, *^ Yingxian Li,^3, *^ Houyu Wang,^2, *^* & *Guangming Zhou^1, *^*

^1^ State Key Laboratory of Radiation Medicine and Protection, School of Radiation Medicine and Protection, Suzhou Medical College of Soochow University, Jiangsu, Suzhou, 215123 P. R. China

^2^ Suzhou Key Laboratory of Nanotechnology and Biomedicine, Institute of Functional Nano & Soft Materials & Collaborative Innovation Center of Suzhou Nano Science and Technology (NANO-CIC), Soochow University, Suzhou 215123, China

^3^ National Key Laboratory of Space Medicine, China Astronaut Research and Training Center, Beijing 100094, China

^4^ Department of Nutrition and Food Hygiene, School of Public Health, Soochow University, Suzhou 215123, China

^5^ Department of Thoracic Surgery, Shanghai Pulmonary Hospital, School of Medicine, Tongji University, Shanghai 200433, China

*Corresponding authors. Email: timyangsh@tongji.edu.cn; yingxianli@aliyun.com; houyuwang@suda.edu.cn; gmzhou@suda.edu.cn

**Inventory of Supporting Information:**

Figures S1-S37

Tables S1-S8


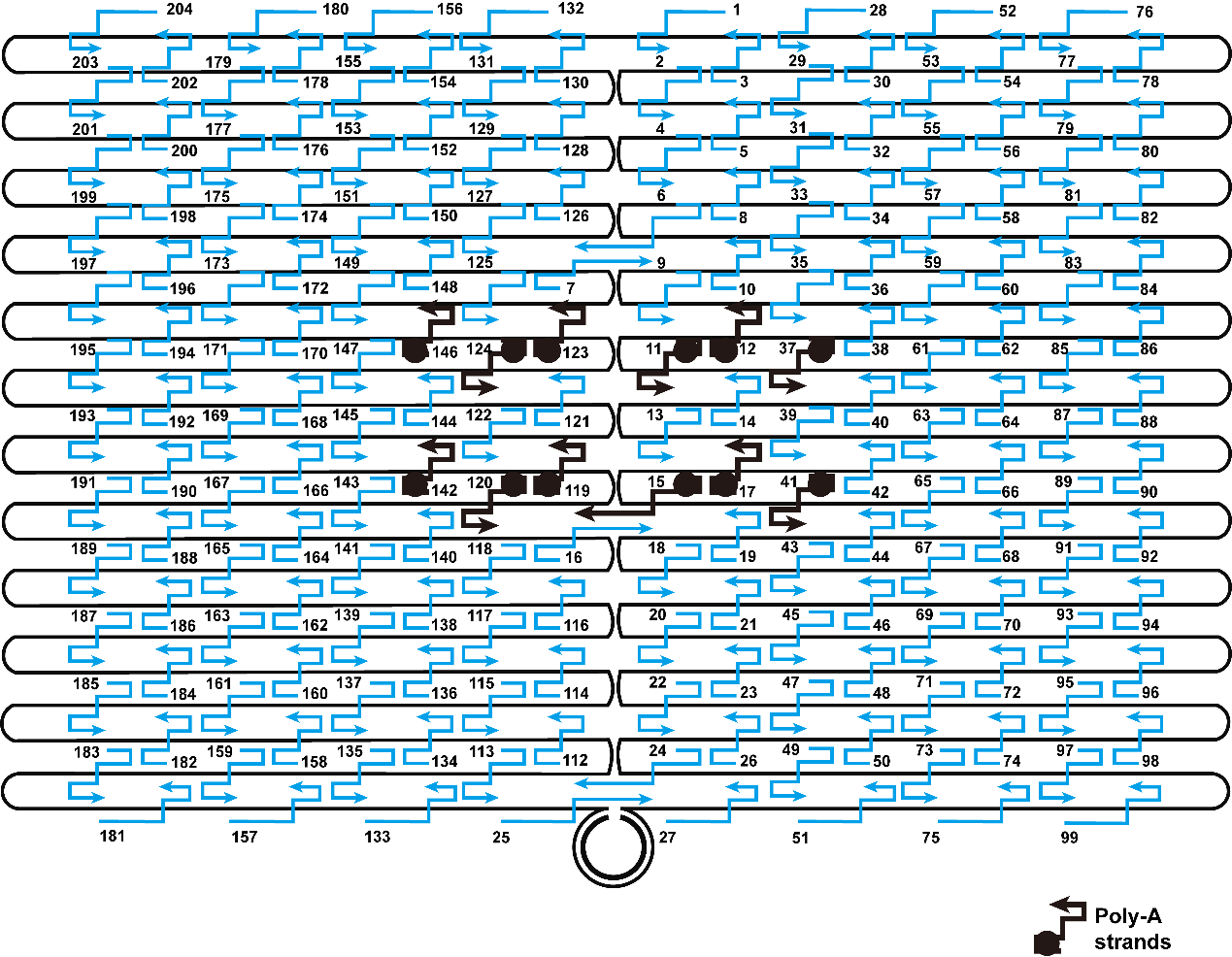


Figure S**1. DNA origami design**. Schematic representation of the blank DNA origami. constructed using the M13 single-stranded DNA scaffold (black) and staple strands with unique sequences and designated positions. To minimize stacking effects during assembly, 24 staple strands from the two broad edges (positions 100–101 and 205–216) were excluded. Poly-A (40As) modifications, indicated by black circles, were introduced for silver nanoparticle attachment.


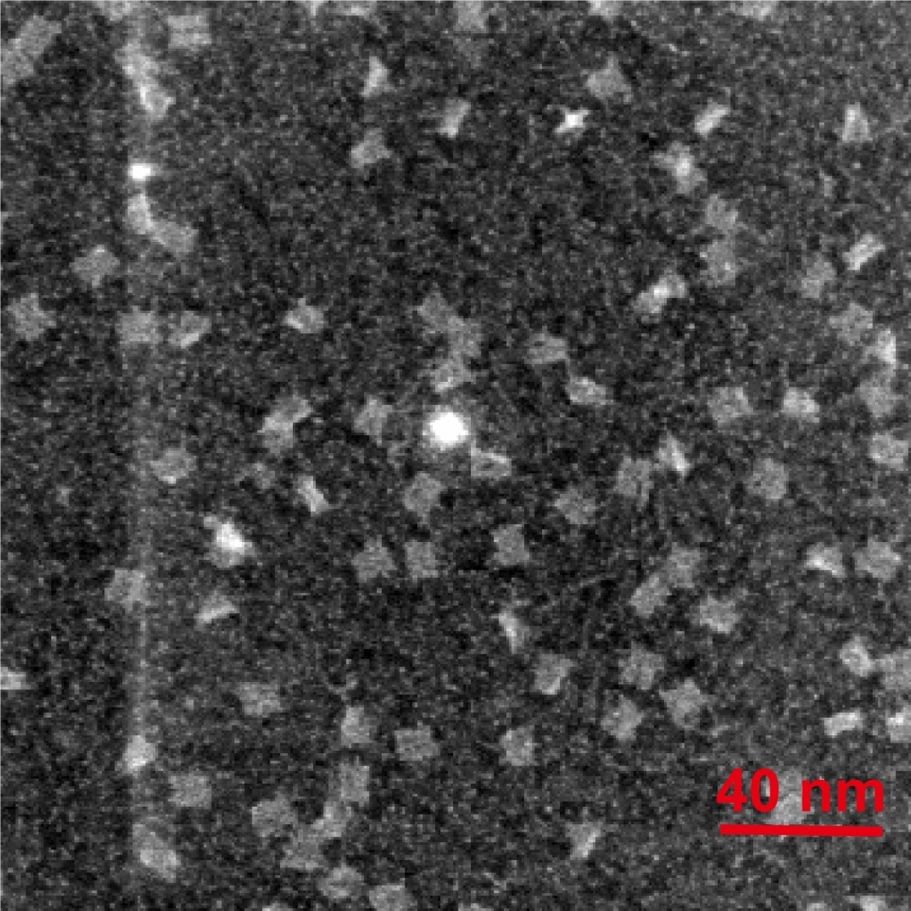


**Figure S**2. TEM images of rectangular DNA origami**.** Scale bar, 40 nm. The imaging experiments were repeated three times with similar results.


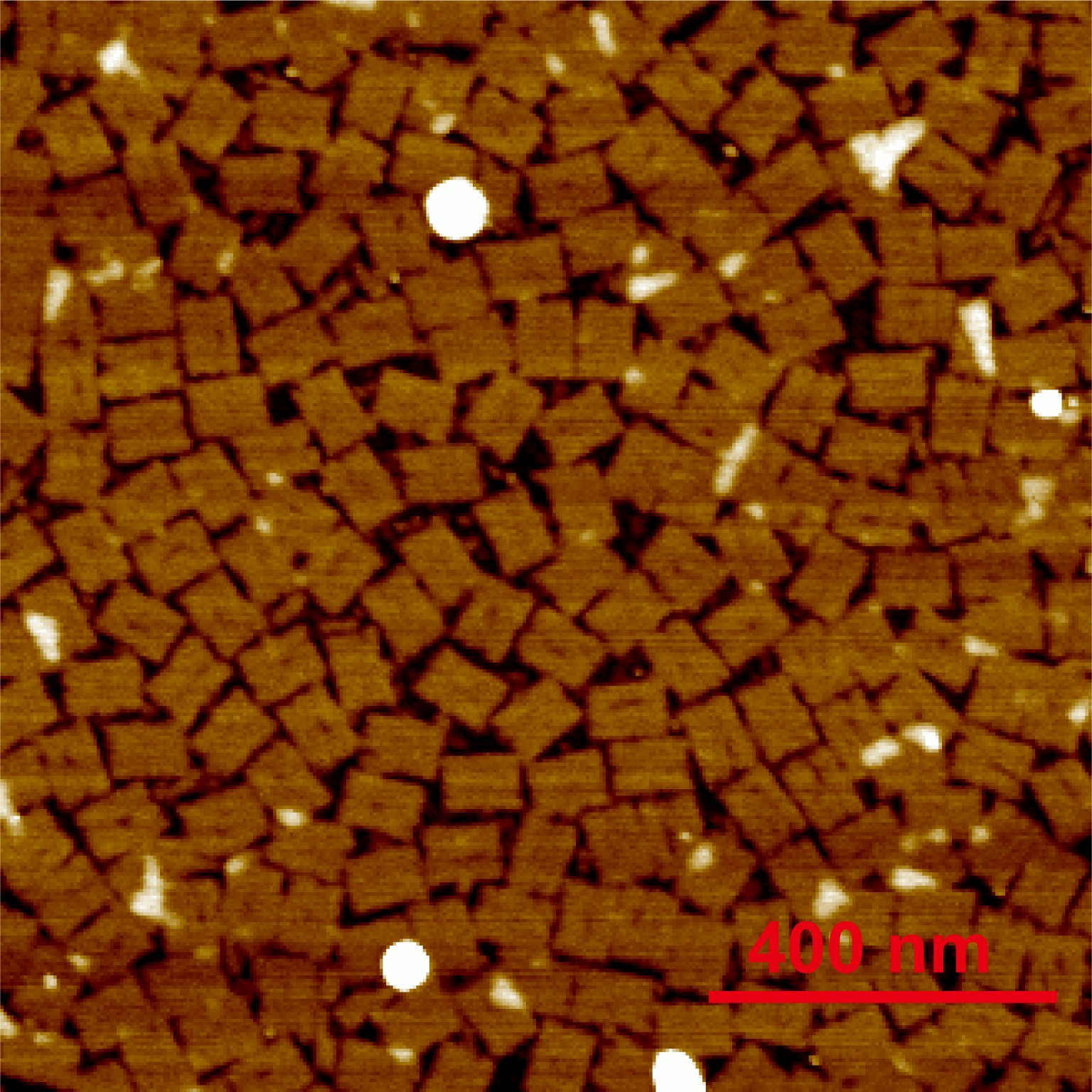


**Figure S**3. AFM images of rectangular DNA origami**.** Scale bar, 400 nm. The imaging experiments were repeated three times with similar results.


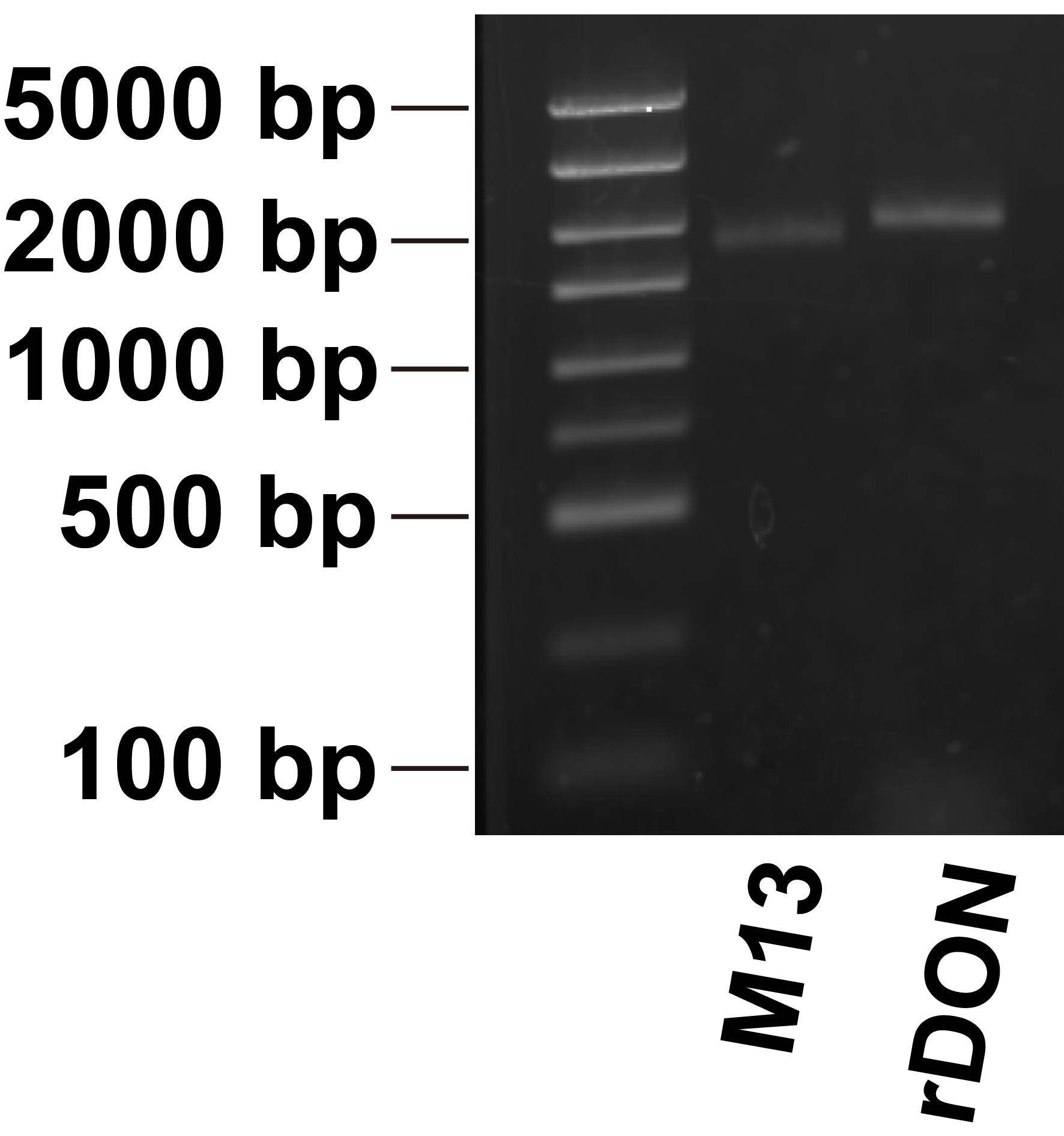


**Figure S**4. AGE images of rectangular DNA origami**.** AGE data for the DNA marker (1 lane), M13 (2 lanes), and rDON (3 lanes). The imaging experiments were repeated three times with similar results.


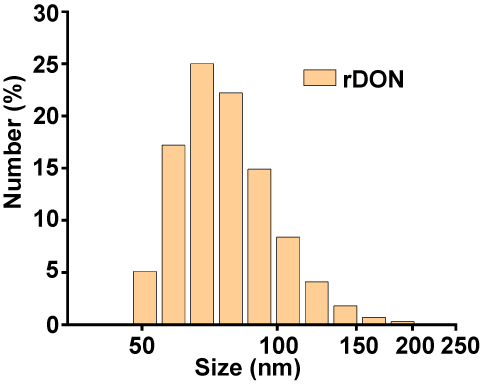


**Figure S**5. DLS data of rectangular DNA origami**.**


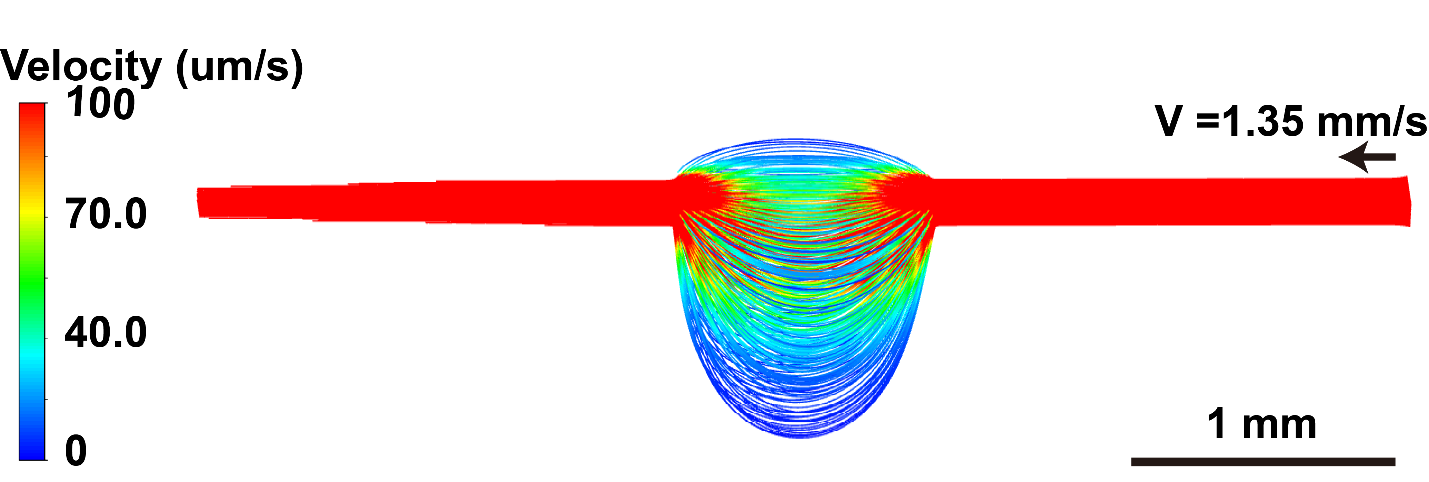


**Figure S**6. The flow profile of the entire flow channel under the microfluidic GD reaction.


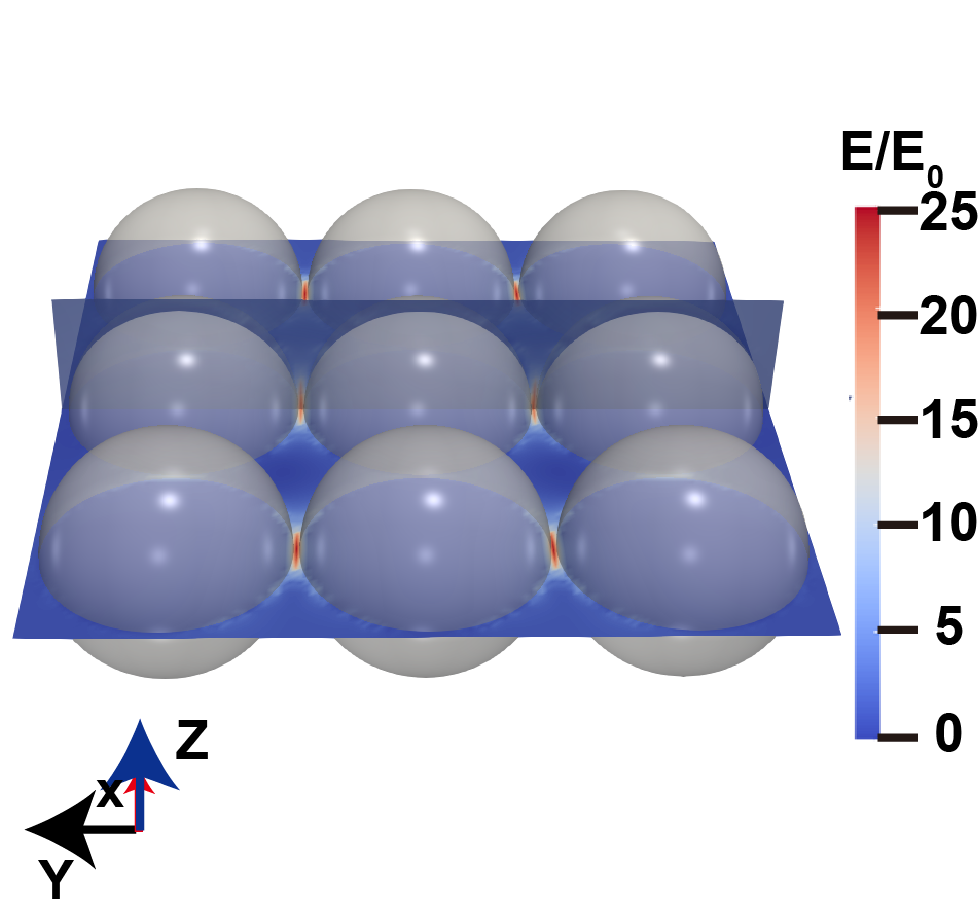


**Figure S**7. FDTD simulation of AgNPs array of the microfluidic system**.** Visualize FDTD simulation results in 3D using ParaView.

**
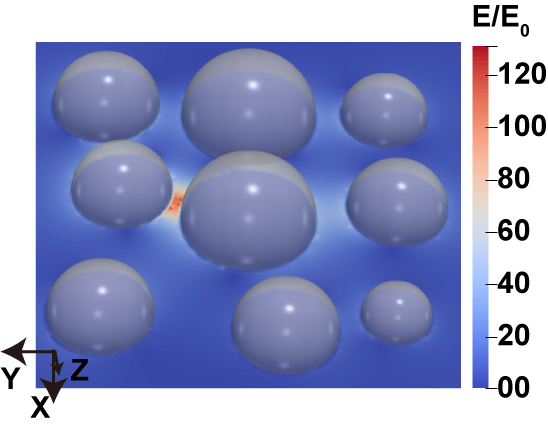
**

**Figure S**8. FDTD simulation of AgNPs array prepared by the traditional bulk system**.** Visualize FDTD simulation results in 3D using ParaView.

**
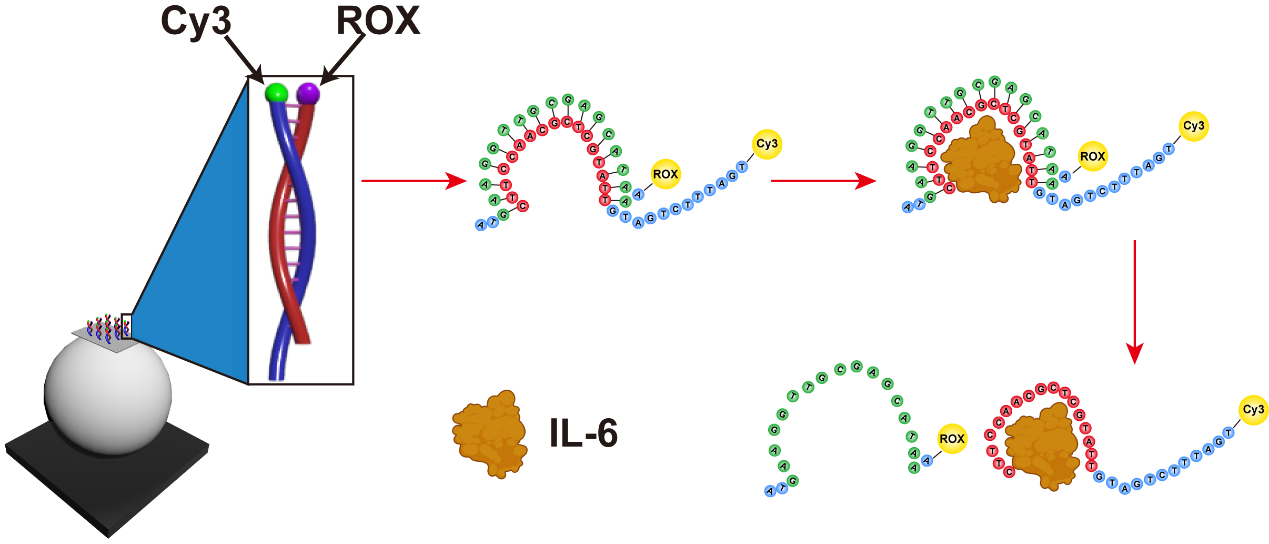
**

**Figure S**9. Schematic diagram of IL-6 detection**.** The IL-6 aptamer modified with ROX dye to the extended chain modified with Cy3 dye on the DNA origami. When IL-6 is recognized, the ROX labelled antenna will dissociate due to the stronger binding force with IL-6, thereby weakening the ROX Raman signal but the Cy3 Raman signal remains unchanged. Quantitative analysis of IL-6 is achieved by quantitatively analyzing the ratio of the ROX Raman characteristic peak to the Cy3 Raman characteristic peak.

**
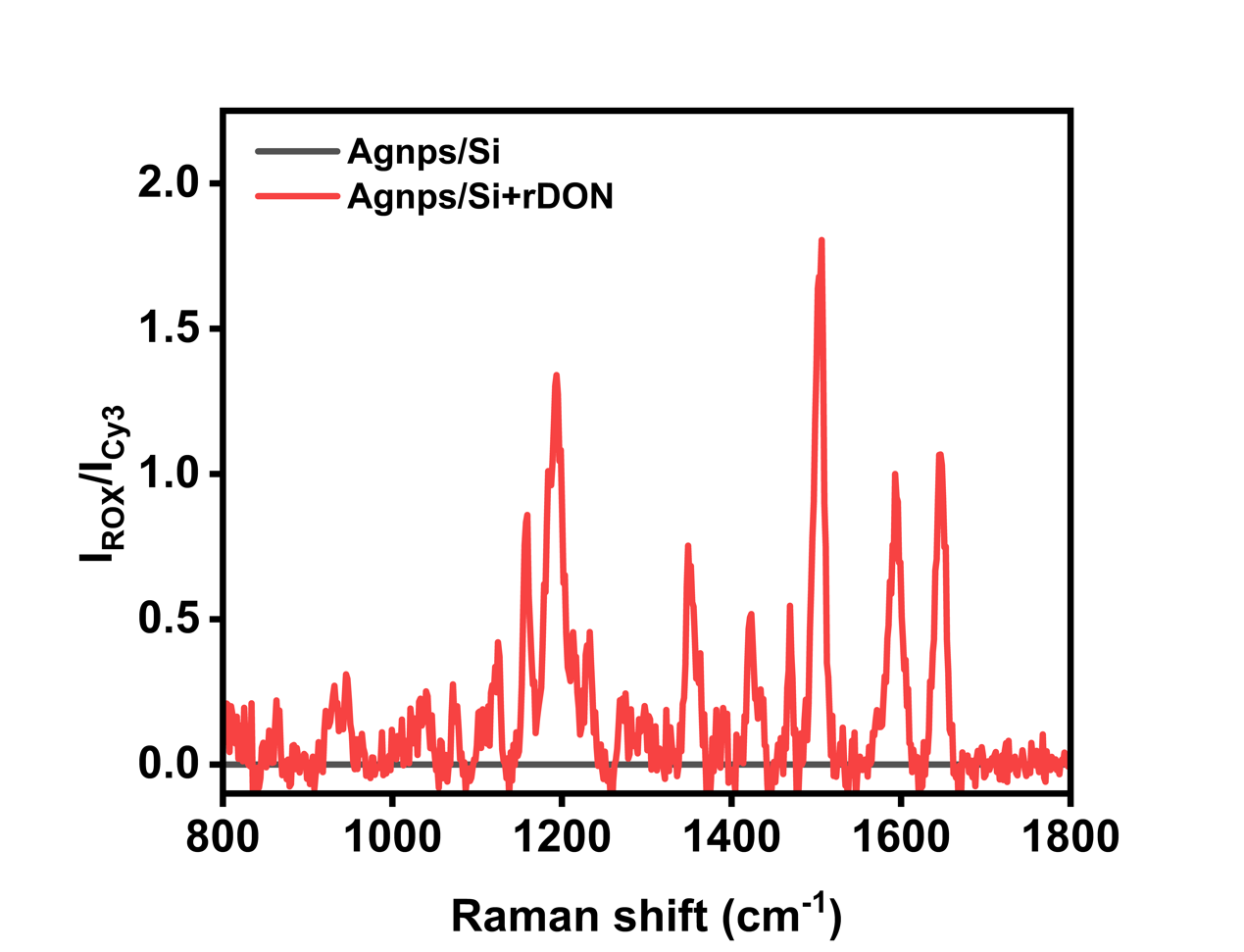
**

**Figure S**10. Raman spectroscopy of u-DON-SERS**.** The successful u-DON-SERS assembly was verified by detecting the Raman peaks of Cy3 (1595 cm^-1^) and ROX (1507 cm^-1^). The portable Raman instrument and the sensor were fixed on a bracket to ensure stable working distance. The excitation wavelength was 785 nm, with medium power and 1 s integration time. Data was exported and processed using Origin software.


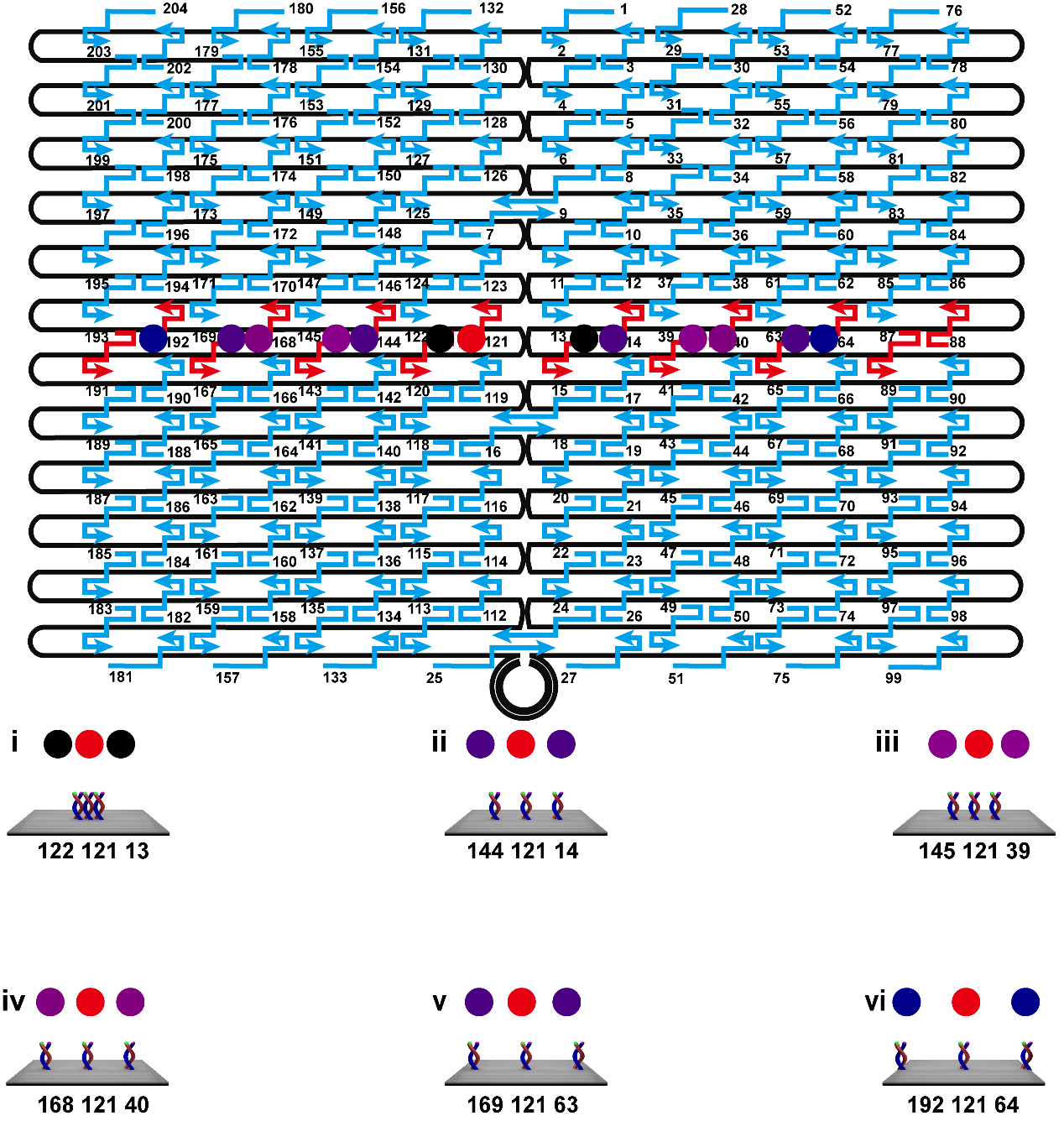


**Figure S**11. Schematic diagram of the design of DNA origami templates with different spacing of recognition antennas**.** The circles of different colors on the origami represent the IL-6 recognition antennas at different sites. The numbers below represent the numbers of the extended chain sites. On the rectangular DNA origami, there are three recognition antennas separated by 0 (i), 1 (ii), 2 (iii), 3 (iv), 4 (v) or 5 (vi) modification sites. The corresponding lateral distances are 5.44 nm, 10.88 nm, 16.32 nm, 21.76 nm, 27.20 nm and 31.64 nm.


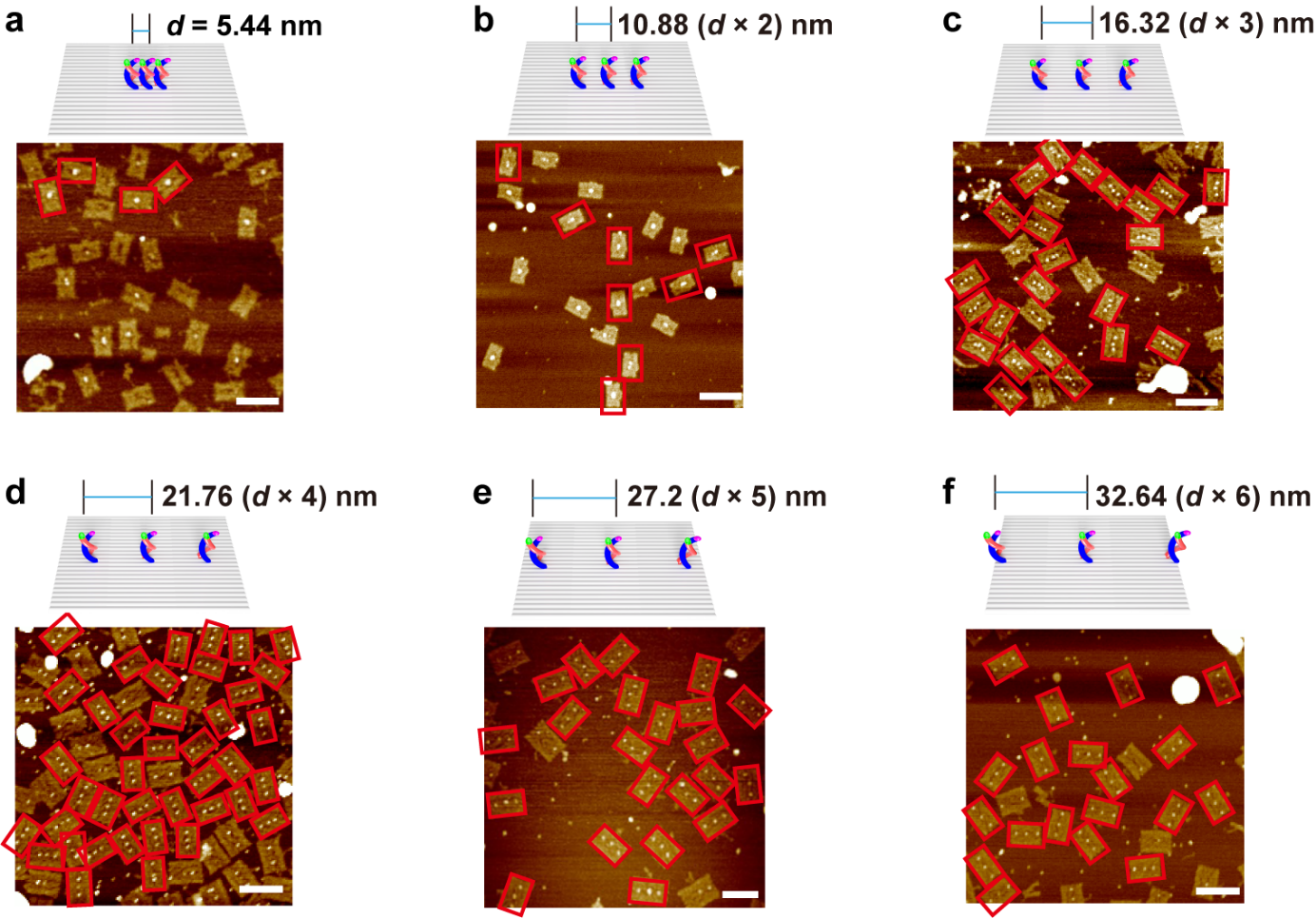


**Figure S**12. **Representative AFM images and height profiles showing antenna spacing of 5.44 (d × 1) nm (a), 10.88 (d × 2) nm (b), 16.32 (d × 3) nm (c), 21.76 (d × 4) nm (d), 27.2 (d × 5) nm (e), and 32.64 (d × 6) nm (f).** d = 5.44 nm, i.e., the minimum spacing of lateral staple extension sites on DNA origami, Scale bar, 100 nm.

**
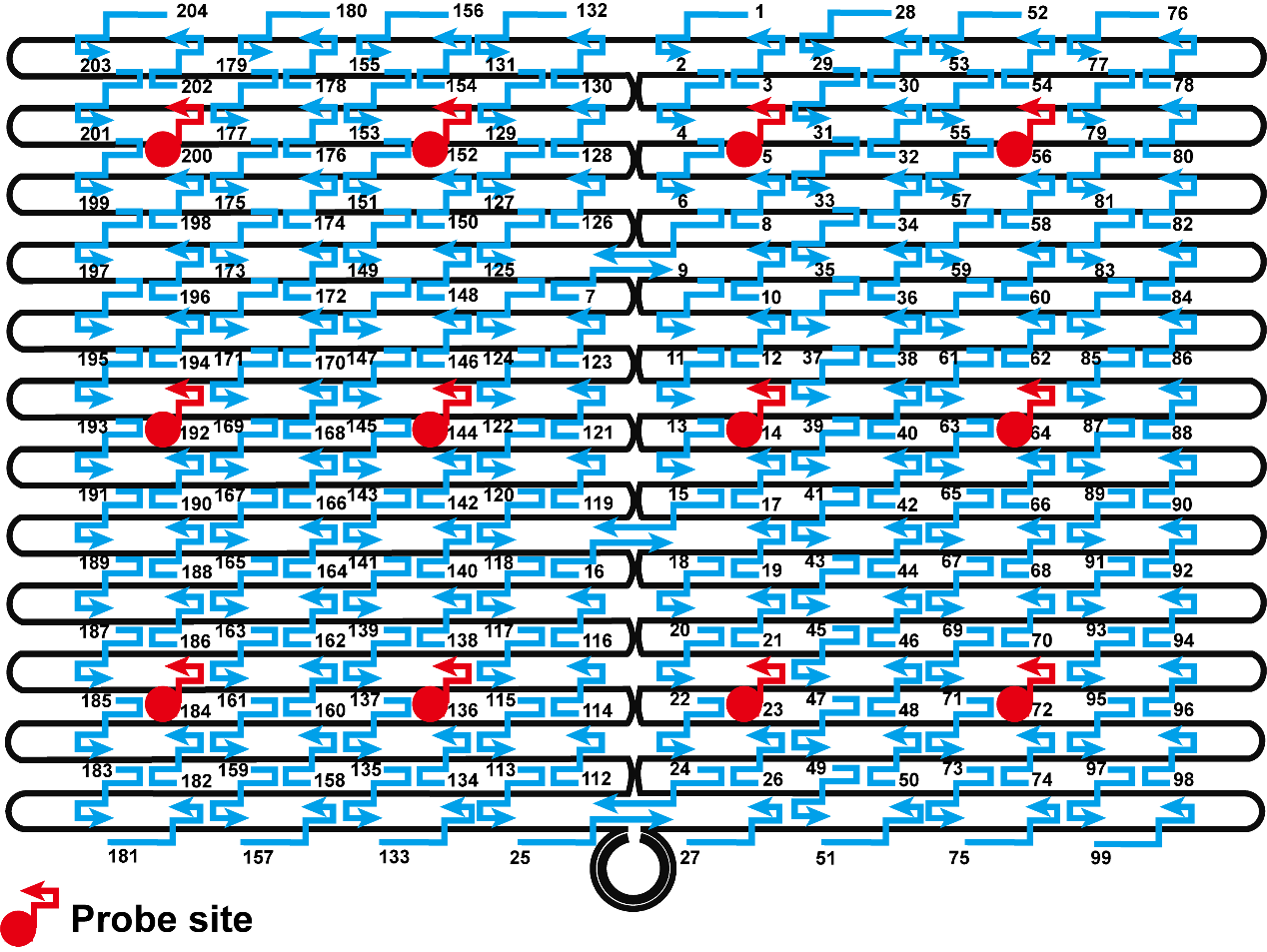
**

**Figure S**13. Schematic diagram of the design of a 3 × 4 antenna array on a rectangular DNA origami**.** The rectangular DNA origami displays a 3 × 4 antennas array with lateral and longitudinal spacings of 21.76 nm and 24 nm, respectively, between adjacent antennas.


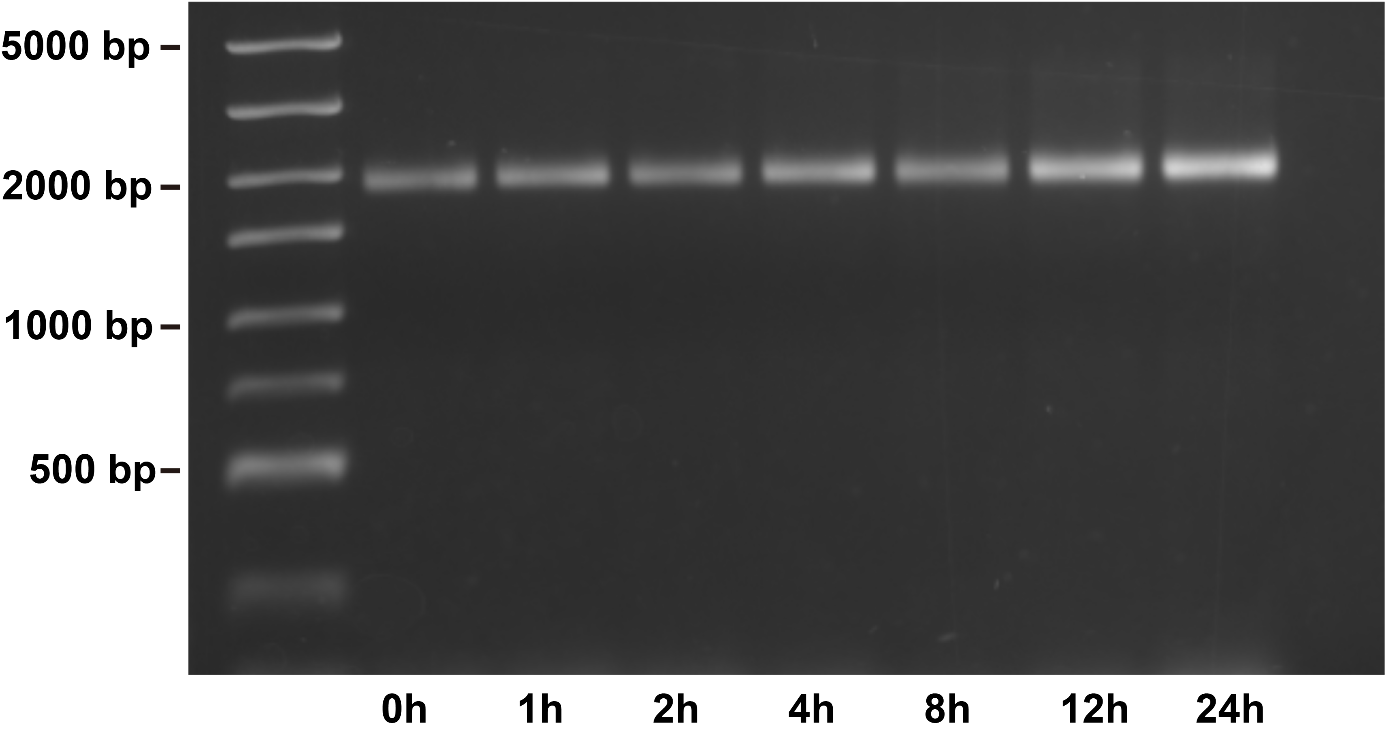


**Figure S**14. **AGE (1%) analysis of 3×4** **antenna array DNA origami incubated in 10% FBS over 0 to 24 hours (bp: base pair).** Data represents one of three independent experiments.

**
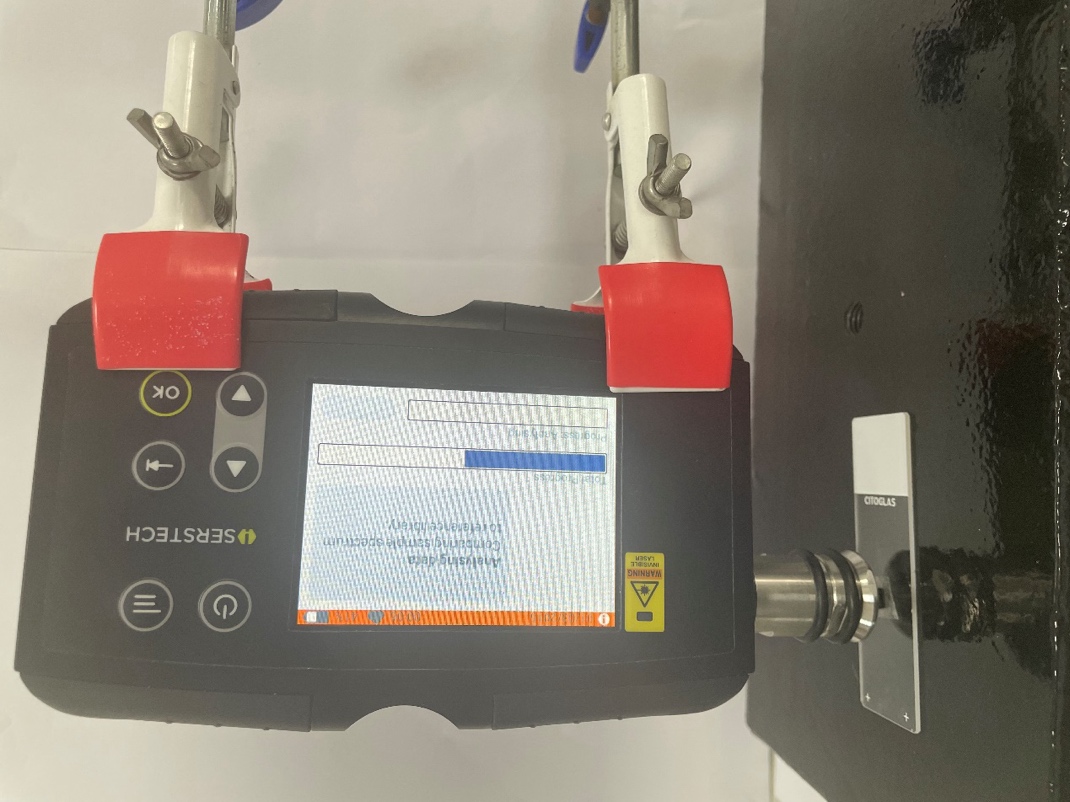
**

**Figure S**15. Actual picture of portable Raman instrument measurement**.**

**
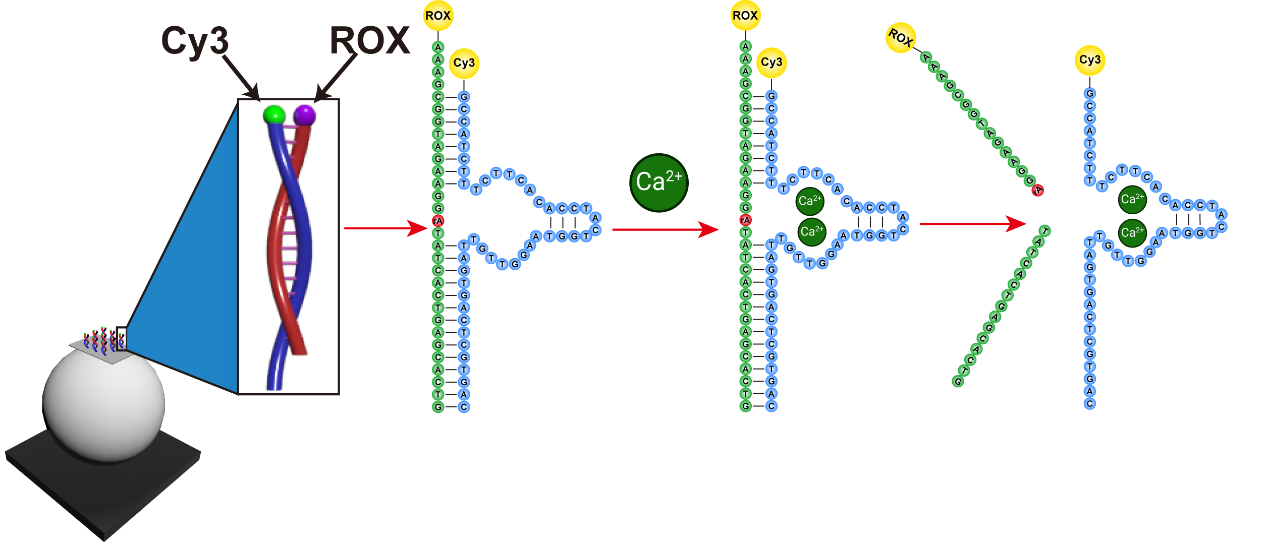
**

**Figure S**16. Schematic diagram of Ca^2+^ detection**.** The Ca^2+^ aptamer modified with ROX dye on the DNA origami is changed into an extended chain modified with Cy3 dye. When recognizing Ca^2+^, the ROX labeled antenna breaks and dissociates due to the interaction with Ca^2+^, thereby weakening the ROX Raman signal, while the Cy3 Raman signal remains unchanged. The quantitative analysis of Ca^2+^ is achieved by quantitatively analyzing the ratio of the ROX Raman characteristic peak to the Cy3 Raman characteristic peak.

**
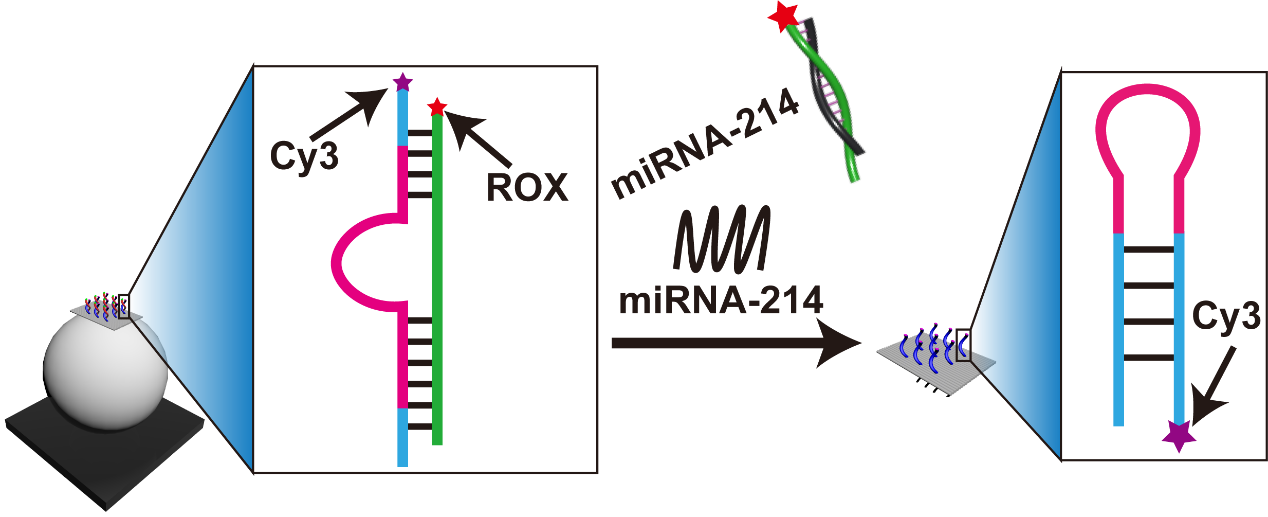
**

**Figure S**17. Schematic diagram of miRNA-214 detection**.** Based on the locked nucleic acid (LNA) strand displacement reaction (LSDR), when miRNA-214 binds, the Y-shaped DNA transforms into a hairpin structure, resulting in a decrease in the ROX Raman signal and an increase in the Cy3 Raman signal.

**
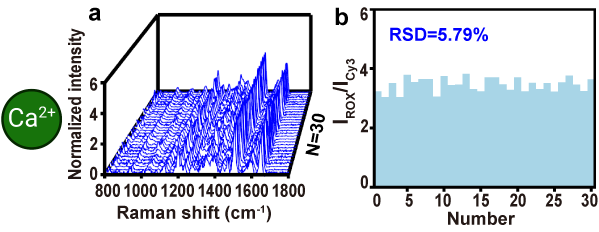
**

**Figure S**18. Repeatability assessment of the Ca^2+^**.** a) SERS mapping spectrum of the u-DON-SERS sensor for detecting Ca²⁺. b) Corresponding ratio values obtained from 30 distinct points within a defined 500 × 500 μm² region. The excitation wavelength was 785 nm, with medium power and 1 s integration time.

**
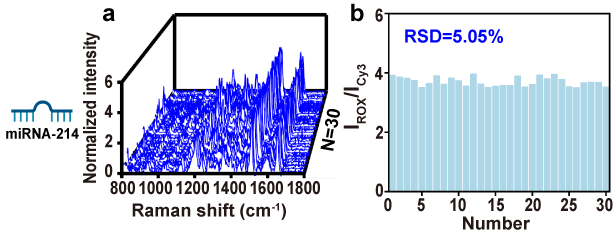
**

**Figure S**19. Repeatability assessment of the miRNA-214**.** a) SERS mapping spectrum of the u-DON-SERS sensor for detecting miRNA-214. b) Corresponding ratio values obtained from 30 distinct points within a defined 500 × 500 μm² region. The excitation wavelength was 785 nm, with medium power and 1 s integration time.

**
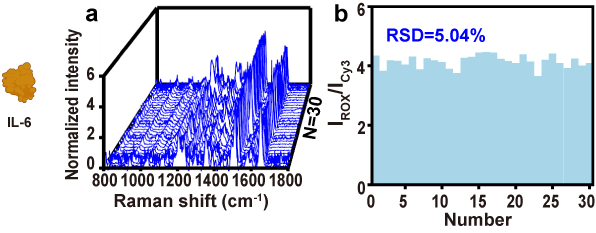
**

**Figure S**20. Repeatability assessment of the IL-6**.** a) SERS mapping spectrum of the u-DON-SERS sensor for detecting IL-6. b) Corresponding ratio values obtained from 30 distinct points within a defined 500 × 500 μm² region. The excitation wavelength was 785 nm, with medium power and 1 s integration time.


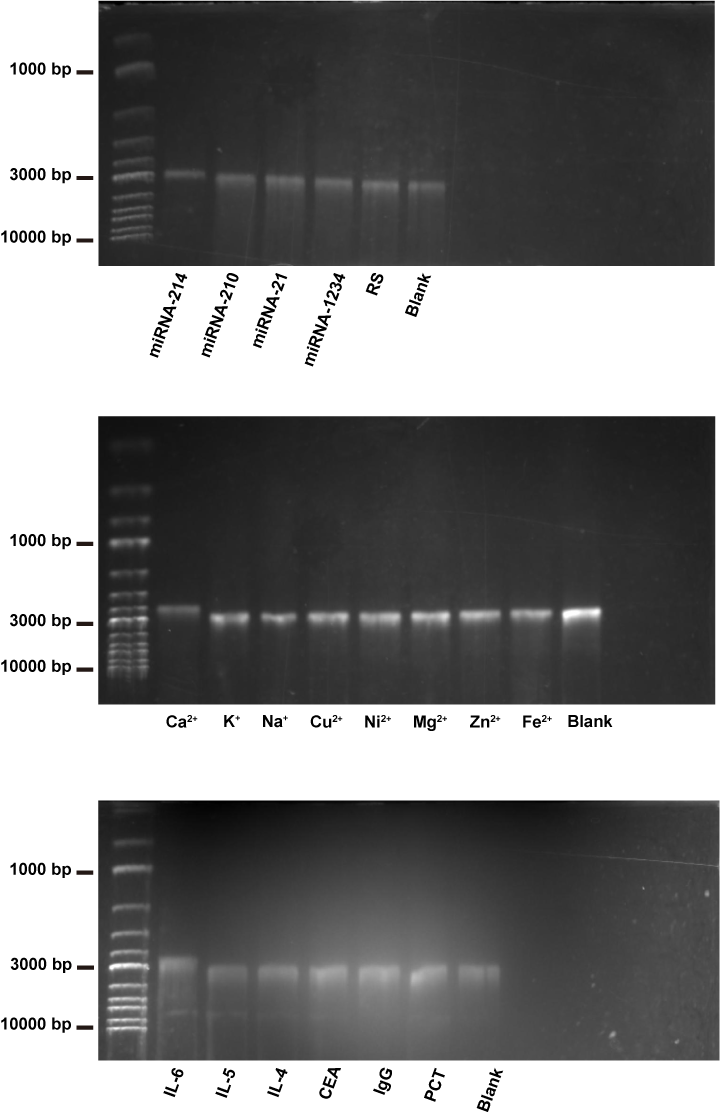


**Figure S21. AGE image showing the effects of different metal ions on the DNA nanostructure.** Upon sufficient treatment with Ca^2^⁺, the aptamer strand undergoes cleavage, resulting in a reduced total base pair (bp) length of the DNA origami structure. In contrast, other tested ions (K⁺, Na⁺, Cu^2^⁺, Ni^2^⁺, Mg^2^⁺, Zn^2^⁺, Fe^2^⁺) caused no noticeable change in the DNA band pattern.


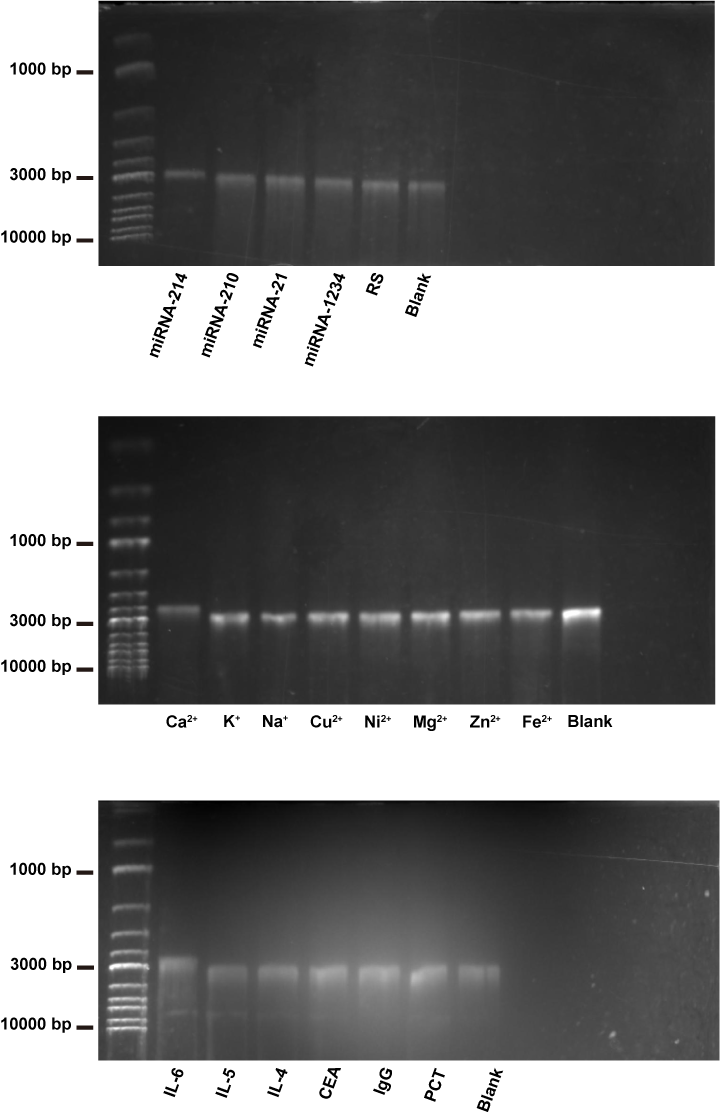


**Figure S22. AGE image showing the effects of different miRNA on the DNA nanostructure.** Upon sufficient treatment with miRNA-214, the aptamer strand undergoes released, resulting in a reduced total base pair (bp) length of the DNA origami structure. In contrast, other miRNAs (miRNA-210, miRNA-21, miRNA-1234), random sequences (RS), and blank controls did not cause significant changes in the DNA band pattern.


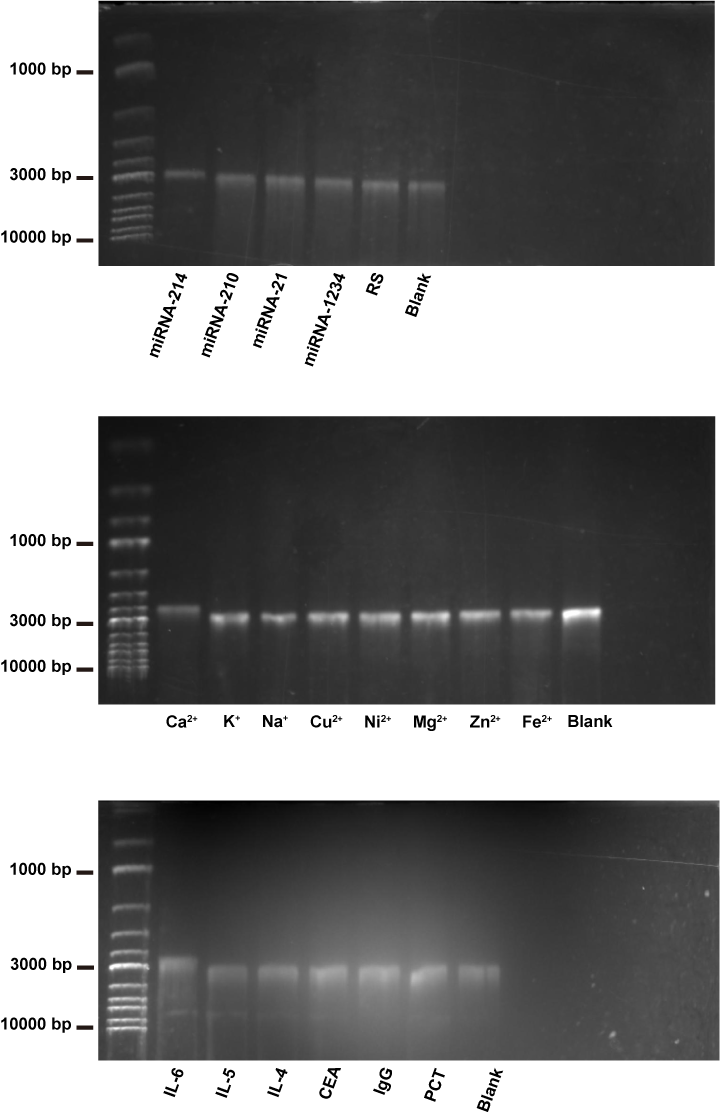


**Figure S23. AGE image showing the effects of different protein biomarkers on the DNA nanostructure.** Upon sufficient treatment with IL-6, the aptamer strand undergoes released, resulting in a reduced total base pair (bp) length of the DNA origami structure. In contrast, other proteins including IL-5, IL-4, CEA, IgG, and PCT, as well as the blank control, showed no observable changes in the DNA band pattern.


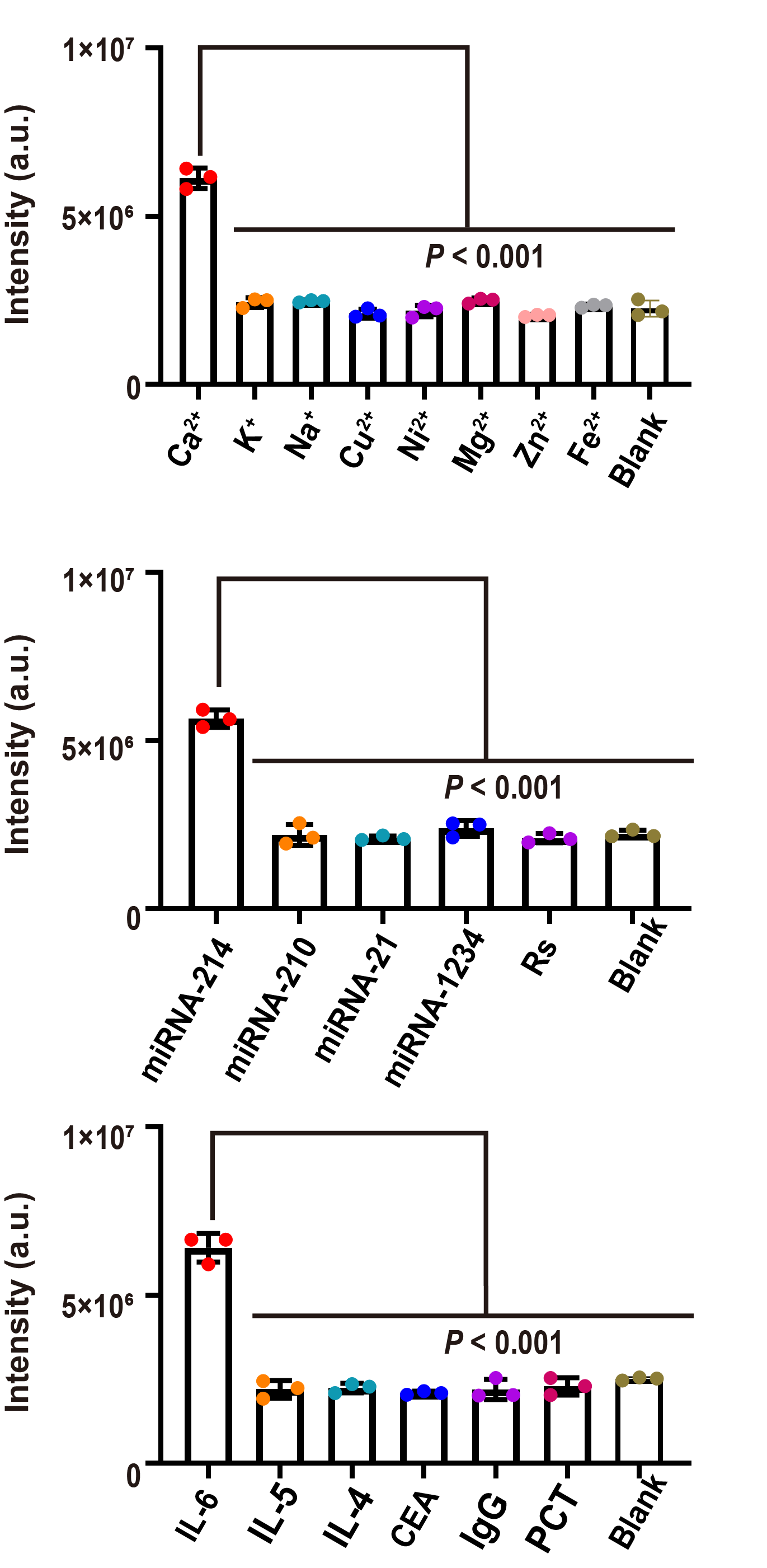


**Figure S24 Effects of different ions on the fluorescence signal of the sensor based on the FRET effect (Ca^2+^, K^+^, Na^+^, Cu^2+^, Ni^2+^, Mg^2+^, Zn^2+^, and Fe^2+^(1 μM each) and blank).** Data are presented as means ± SD. Error bars = SD. Statistical significance was determined using one-way ANOVA with Dunnett's test.


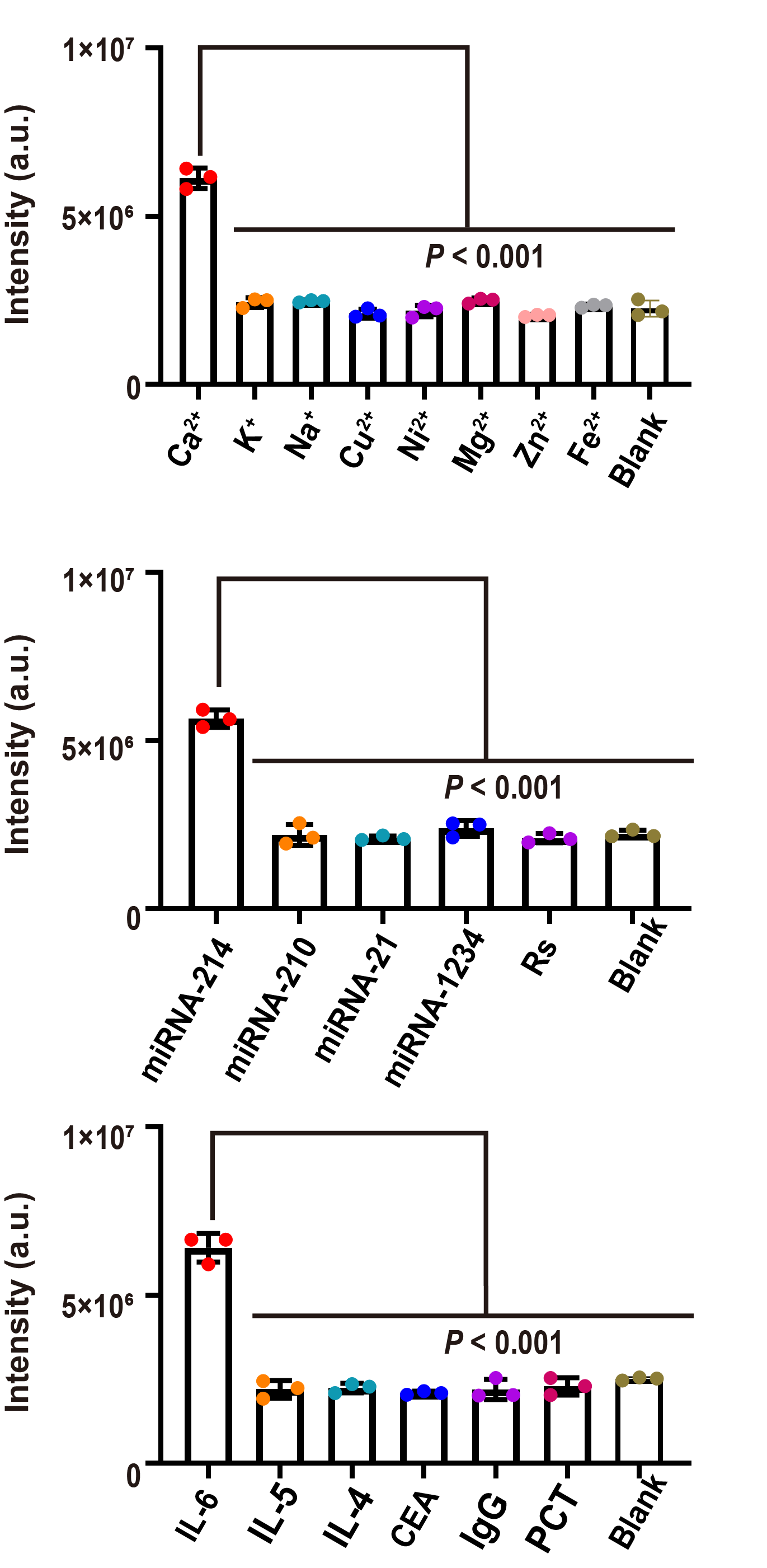


**Figure S25. Effects of different miRNA on the fluorescence signal of the sensor based on the FRET effect (miRNA-214, miRNA-210, miRNA-21, miRNA-1234, random sequence RS (10 pM each) and blank).** Data are presented as means ± SD. Error bars = SD. Statistical significance was determined using one-way ANOVA with Dunnett's test.


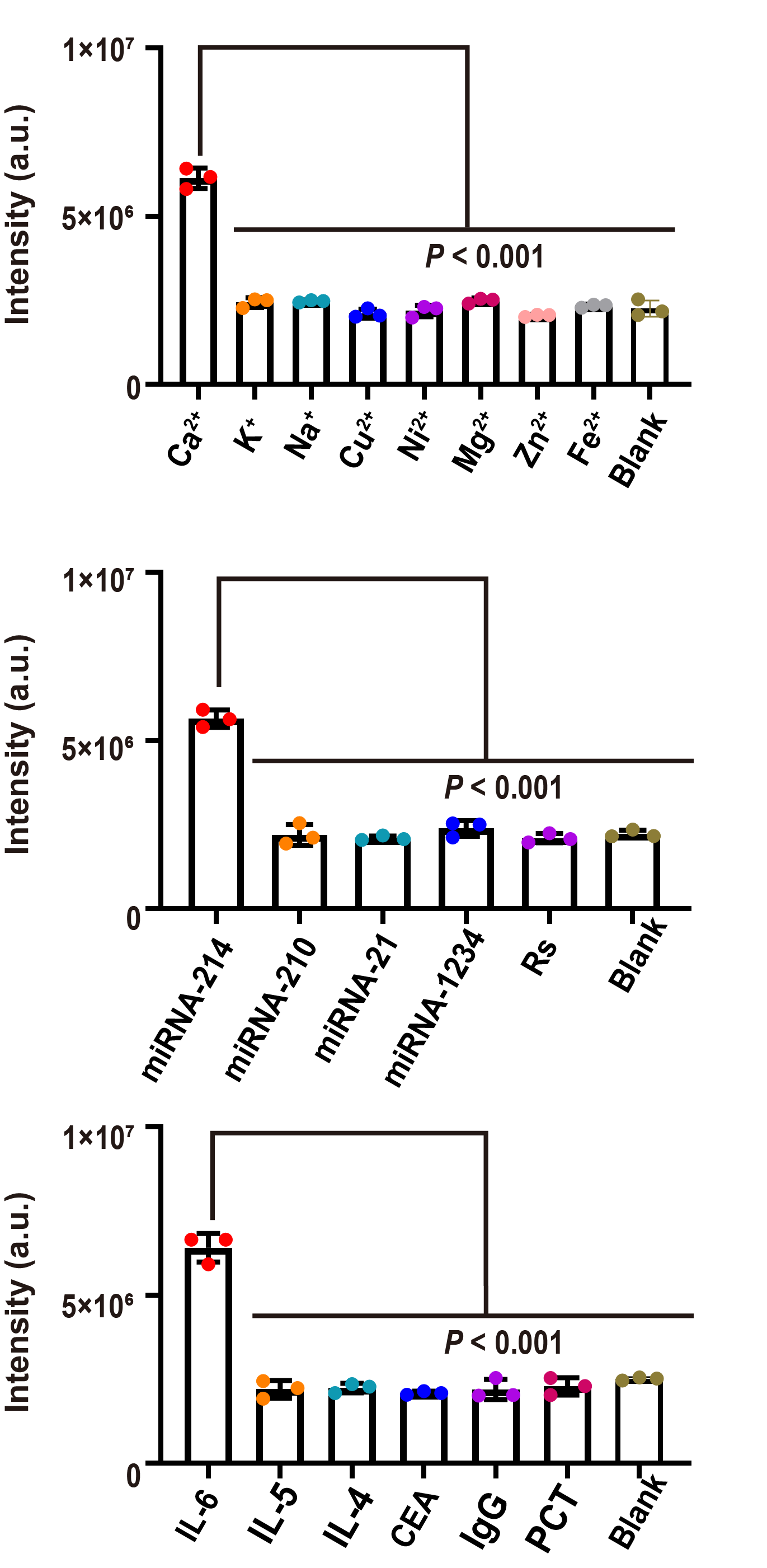


**Figure S26. Effects of different proteins on the fluorescence signal of the sensor based on the FRET effect (IL-6, IL-5, IL-4, CEA, IgG, PCT (100 pg/mL each) and blank).** Data are presented as means ± SD. Error bars = SD. Statistical significance was determined using one-way ANOVA with Dunnett's test.


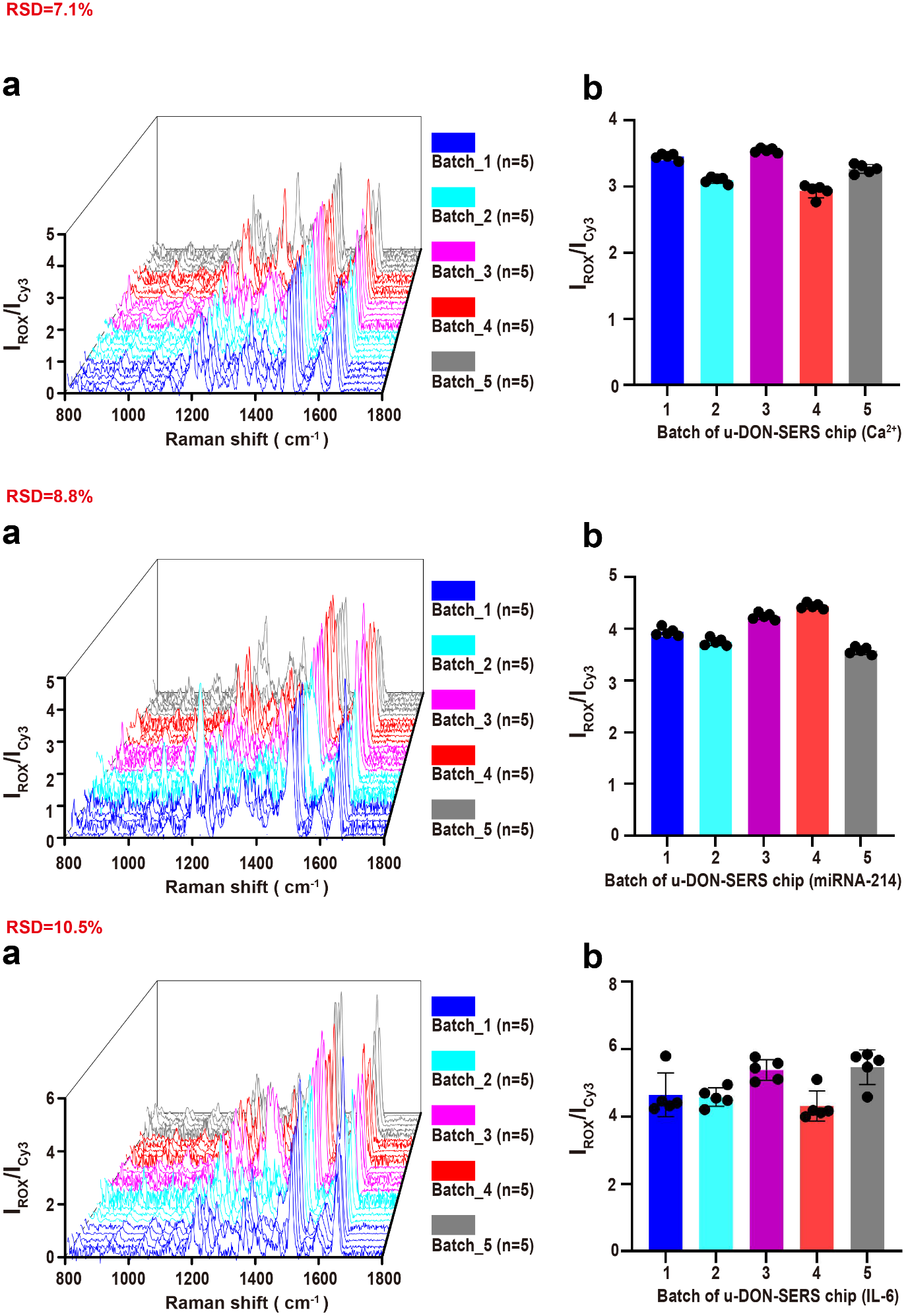


**Figure S27. Reproducibility in the course of SERS signal measurement for five batches of u-DON-SERS chips (Ca^2+^ detection).** (a) SERS spectra for five batches of u-DON-SERS chips and corresponding (b) ratiometric signals (I_ROX_/I_Cy3_) values with different batches.


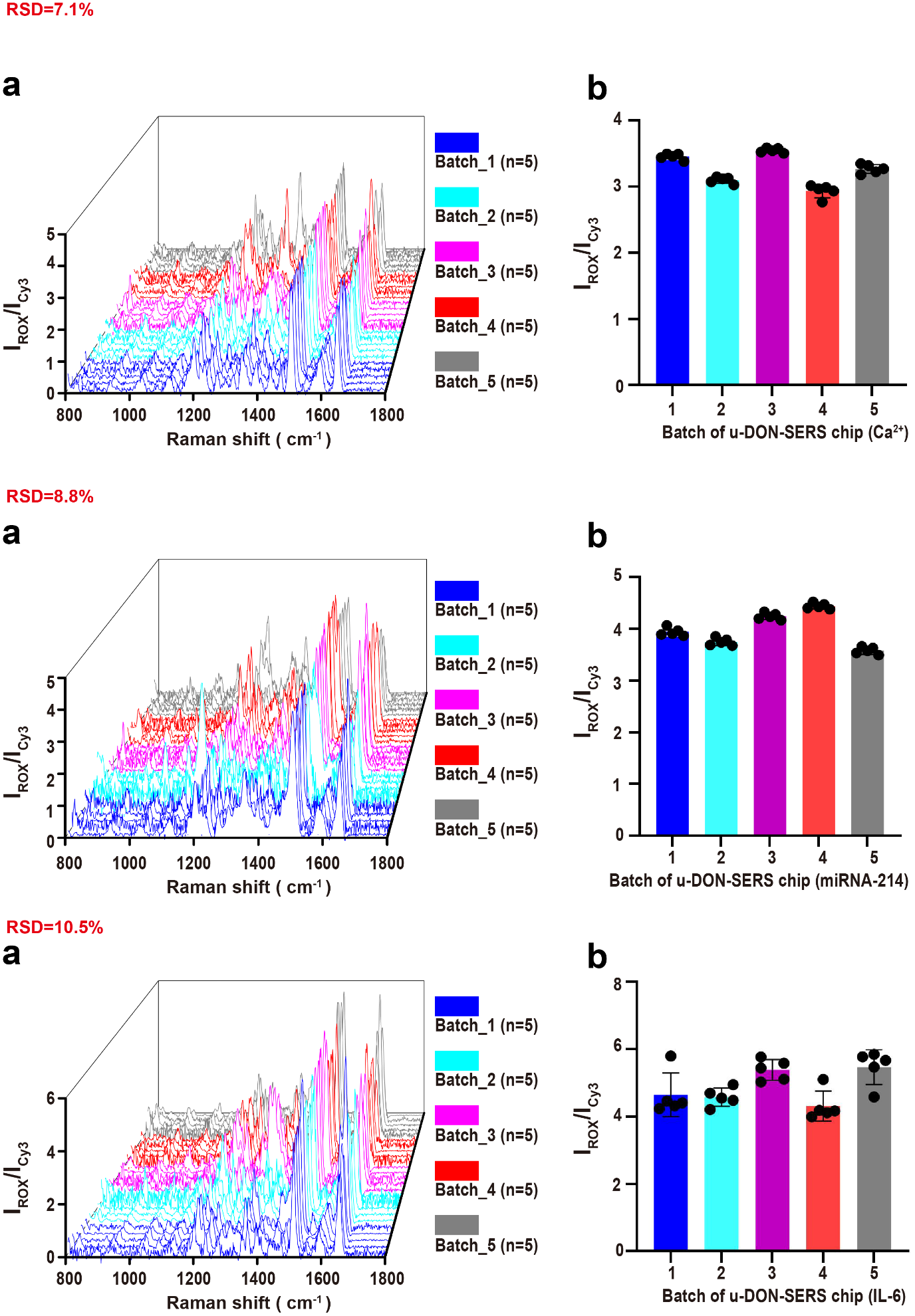


**Figure S28. Reproducibility in the course of SERS signal measurement for five batches of u-DON-SERS chips (miRNA-214 detection).** (a) SERS spectra for five batches of u-DON-SERS chips and corresponding (b) ratiometric signals (I_ROX_/I_Cy3_) values with different batches.


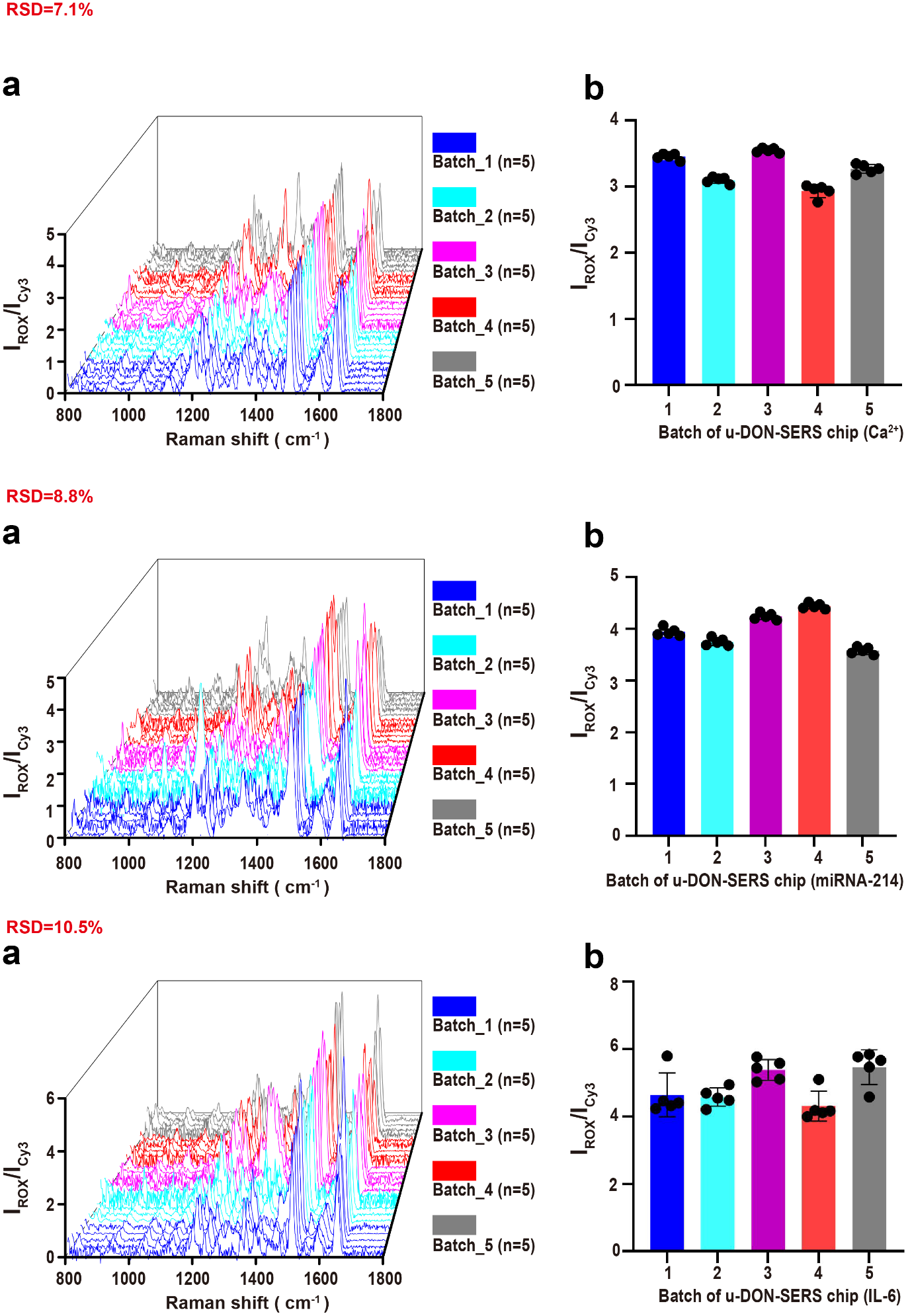


**Figure S29. Reproducibility in the course of SERS signal measurement for five batches of u-DON-SERS chips (IL-6 detection).** (a) SERS spectra for five batches of u-DON-SERS chips and corresponding (b) ratiometric signals (I_ROX_/I_Cy3_) values with different batches.


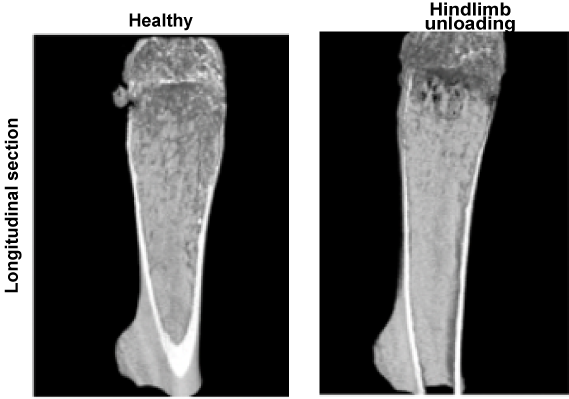


**Figure S**30. Bone phenotype analysis of heathy and hindlimb unloading mice**.** Data presents one of three independent experiments.


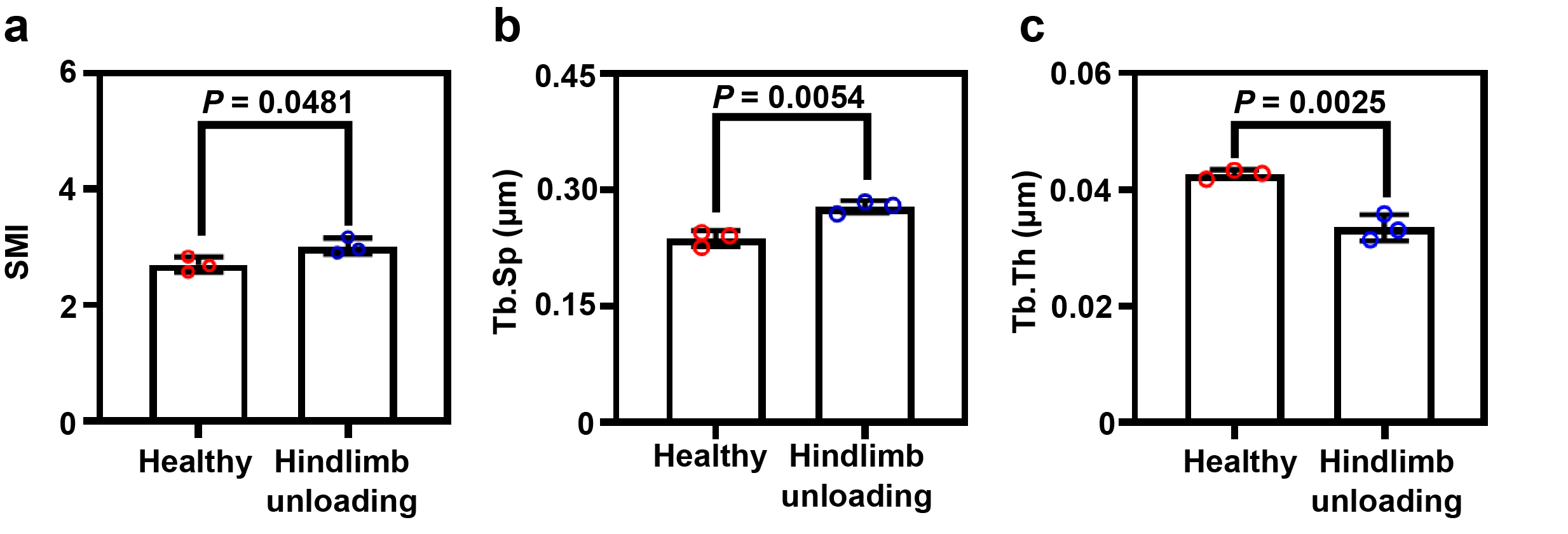


**Figure S**31. Quantitative analysis of micro-CT measurements**.** a) Quantitative micro-CT measurements of structural model index (SMI) in healthy and hindlimb unloading mice. b) Quantitative micro-CT measurements of rabecular separation (Tb.Sp) in healthy and hindlimb unloading mice. c) Quantitative micro-CT measurements of trabecular thickness (Tb.Th) in healthy and hindlimb unloading mice. The data was presented as the means ± SDs. Error bars = SD. *P* values in (a), (b), and (c) were calculated *via* an unpaired two-tailed t test. *P* < 0.05 were shown.


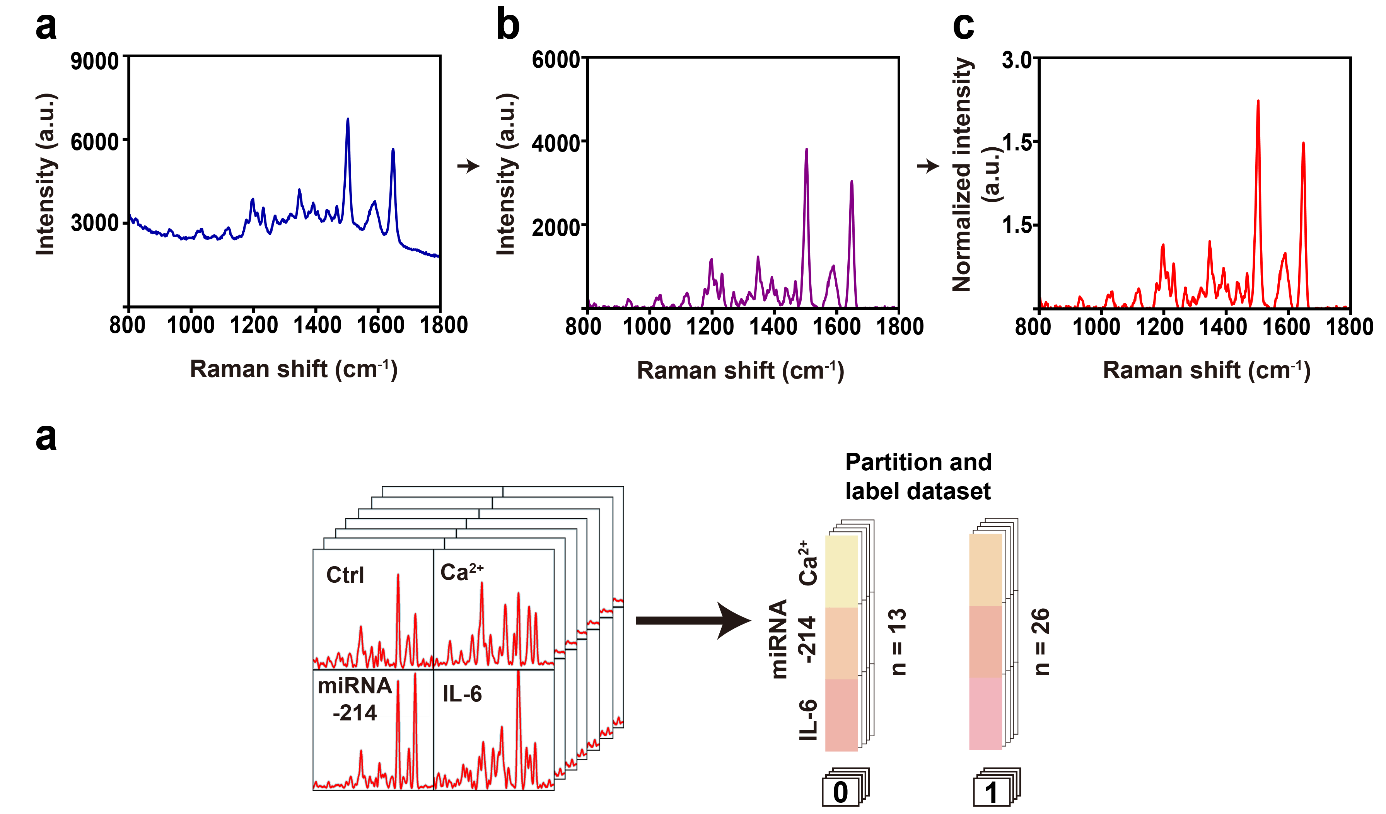


**Figure S**32. Preprocessing of SERS spectral data**.** (a) Raw spectrum before processing. (b) Baseline correction using linear interpolation between negative peaks. (c) Normalized spectrum based on the Cy3 Raman characteristic peak at 1595 cm⁻^1^.


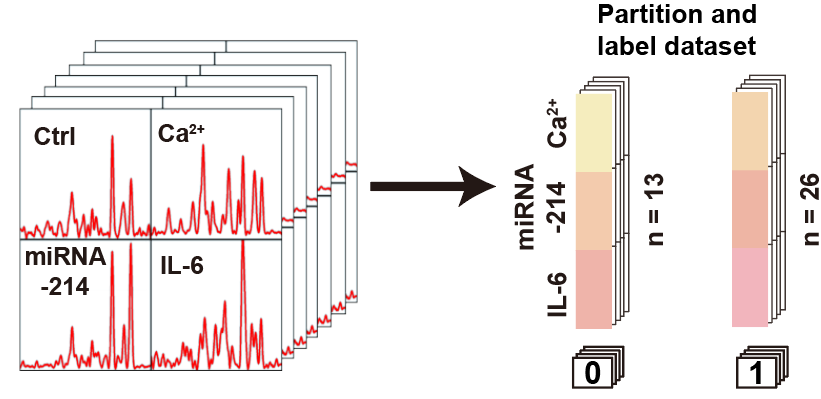


**Figure S**33. **Compiled spectral dataset, summarizing concentrations of serum Ca^2^⁺, miRNA-214, and IL-6, and categorizing samples into healthy (group “0”) or bone loss (group “1”).**


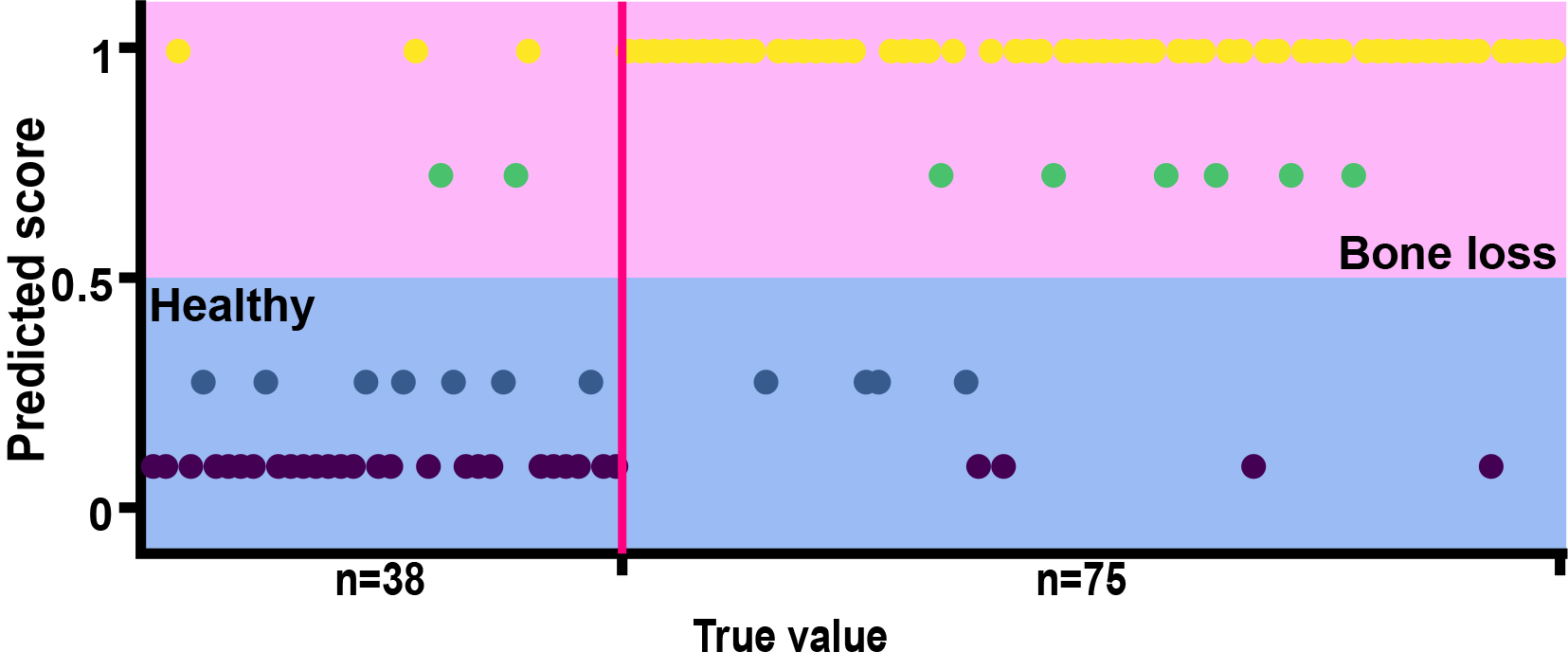


**Figure S**34. **DT predicted scores of all test samples.**


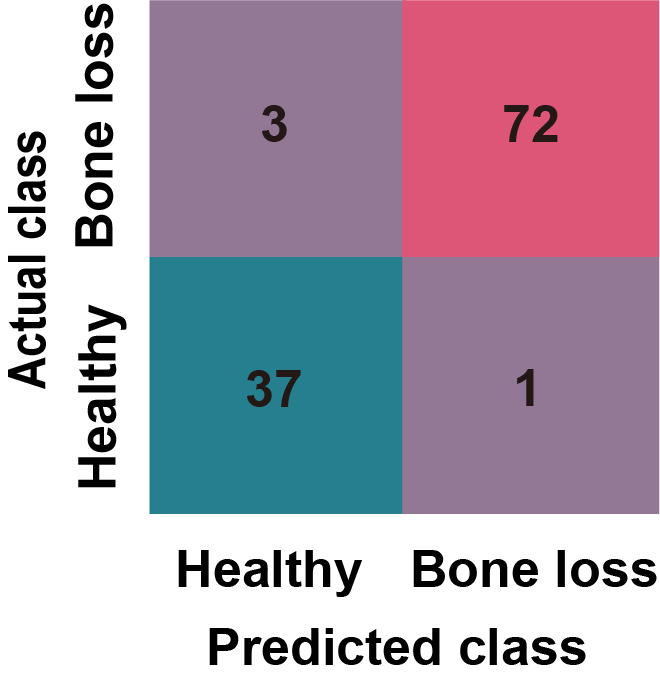


**Figure S35. The confusion matrix of the CNN model.**

**
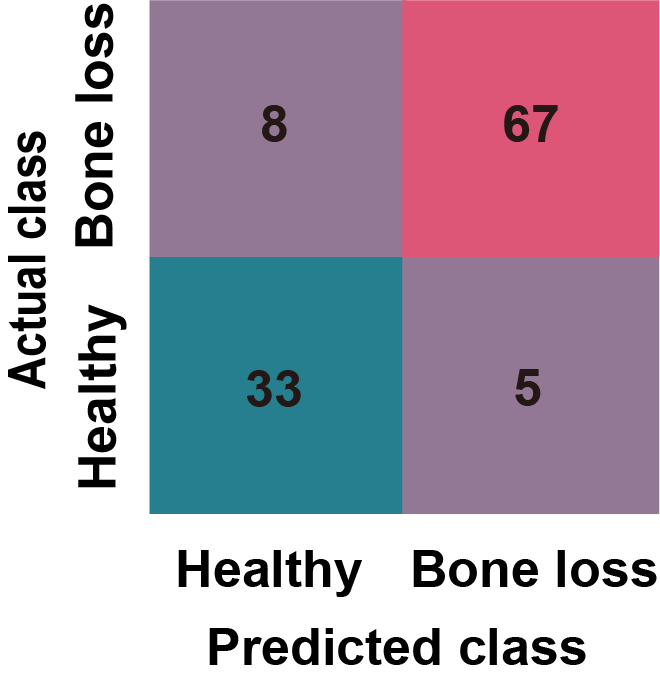
**

**Figure S36 The confusion matrix of the DT model.**


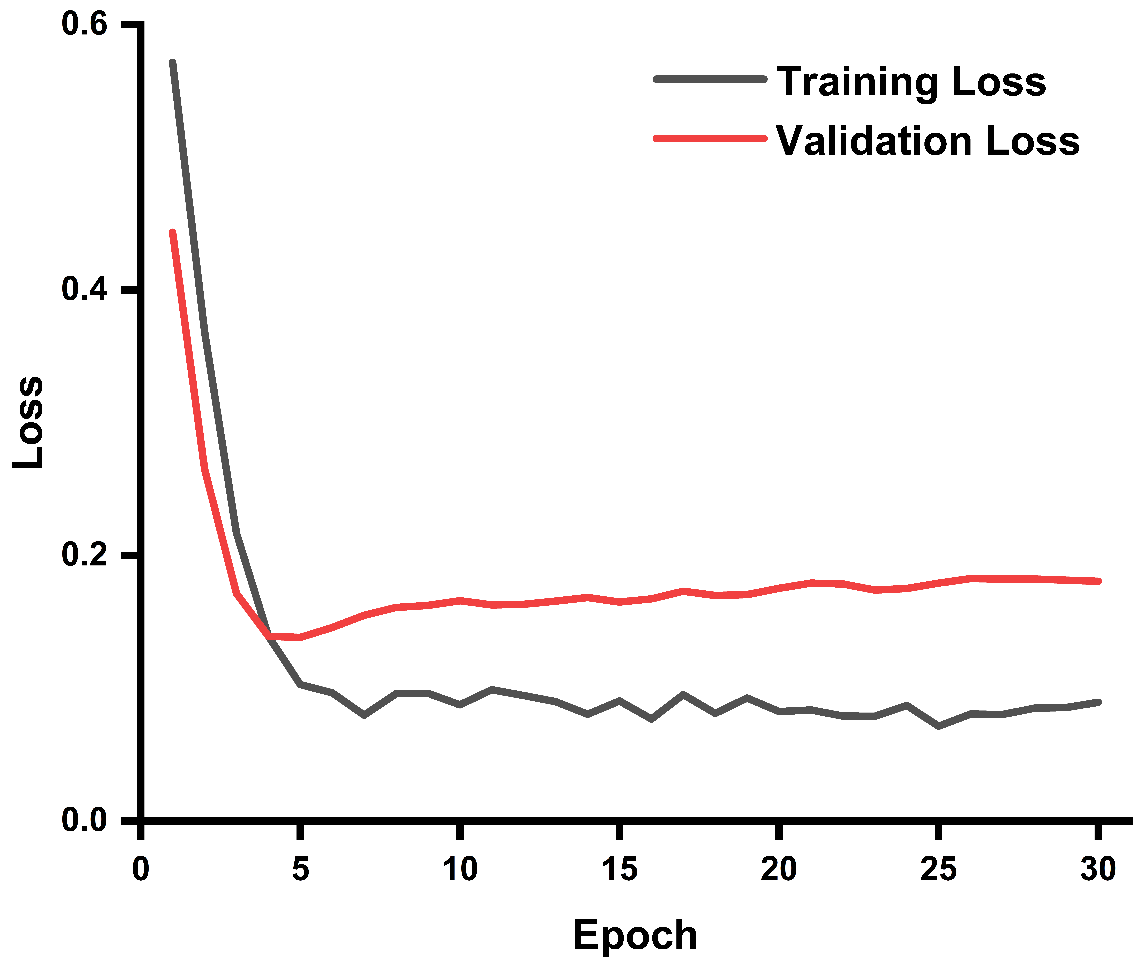


**Figure S37. Learning curve during neural network training.**

Table S1. Rectangular DNA origami tile and the corresponding staple strands (left to right 5’-3’)**.**

| **Name** | **DNA Sequence** |
| --- | --- |
| 1 | CAAGCCCAATAGGAAC CCATGTACAAACAGTT |
| 2 | AATGCCCCGTAACAGT GCCCGTATCTCCCTCA |
| 3 | TGCCTTGACTGCCTAT TTCGGAACAGGGATAG |
| 4 | GAGCCGCCCCACCACCGGAACCGCGACGGAAA |
| 5 | AACCAGAGACCCTCAGAACCGCCAGGGGTCAG |
| 6 | TTATTCATAGGGAAGG TAAATATT CATTCAGT |
| 7 | CATAACCCGAGGCATA GTAAGAGC TTTTTAAG |
| 8 | ATTGAGGGTAAAGGTG AATTATCAATCACCGG |
| 9 | AAAAGTAATATCTTAC CGAAGCCCTTCCAGAG |
| 10 | GCAATAGCGCAGATAG CCGAACAATTCAACCG |
| 11 | CCTAATTTACGCTAAC GAGCGTCTAATCAATA |
| 12 | TCTTACCAGCCAGTTA CAAAATAAATGAAATA |
| 13 | ATCGGCTGCGAGCATG TAGAAACCTATCATAT |
| 14 | CTAATTTATCTTTCCT TATCATTCATCCTGAA |
| 15 | GCGTTATAGAAAAAGC CTGTTTAG AAGGCCGG |
| 16 | GCTCATTTTCGCATTA AATTTTTG AGCTTAGA |
| 17 | AATTACTACAAATTCT TACCAGTAATCCCATC |
| 18 | TTAAGACGTTGAAAAC ATAGCGATAACAGTAC |
| 19 | TAGAATCCCTGAGAAG AGTCAATAGGAATCAT |
| 20 | CTTTTACACAGATGAA TATACAGTAAACAATT |
| 21 | TTTAACGTTCGGGAGA AACAATAATTTTCCCT |
| 22 | CGACAACTAAGTATTA GACTTTACAATACCGA |
| 23 | GGATTTAGCGTATTAA ATCCTTTGTTTTCAGG |
| 24 | ACGAACCAAAACATCG CCATTAAA TGGTGGTT |
| 25 | GAACGTGGCGAGAAAG GAAGGGAA CAAACTAT |
| 26 | TAGCCCTACCAGCAGA AGATAAAAACATTTGA |
| 27 | CGGCCTTGCTGGTAAT ATCCAGAACGAACTGA |
| 28 | CTCAGAGCCACCACCC TCATTTTCCTATTATT |
| 29 | CTGAAACAGGTAATAA GTTTTAACCCCTCAGA |
| 30 | AGTGTACTTGAAAGTA TTAAGAGGCCGCCACC |
| 31 | GCCACCACTCTTTTCA TAATCAAACCGTCACC |
| 32 | GTTTGCCACCTCAGAG CCGCCACCGATACAGG |
| 33 | GACTTGAGAGACAAAA GGGCGACAAGTTACCA |
| 34 | AGCGCCAACCATTTGG GAATTAGATTATTAGC |
| 35 | GAAGGAAAATAAGAGC AAGAAACAACAGCCAT |
| 36 | GCCCAATACCGAGGAA ACGCAATAGGTTTACC |
| 37 | ATTATTTAACCCAGCT ACAATTTTCAAGAACG |
| 38 | TATTTTGCTCCCAATC CAAATAAGTGAGTTAA |
| 39 | GGTATTAAGAACAAGA AAAATAATTAAAGCCA |
| 40 | TAAGTCCTACCAAGTA CCGCACTCTTAGTTGC |
| 41 | ACGCTCAAAATAAGAA TAAACACCGTGAATTT |
| 42 | AGGCGTTACAGTAGGG CTTAATTGACAATAGA |
| 43 | ATCAAAATCGTCGCTA TTAATTAACGGATTCG |
| 44 | CTGTAAATCATAGGTC TGAGAGACGATAAATA |
| 45 | CCTGATTGAAAGAAAT TGCGTAGACCCGAACG |
| 46 | ACAGAAATCTTTGAAT ACCAAGTTCCTTGCTT |
| 47 | TTATTAATGCCGTCAA TAGATAATCAGAGGTG |
| 48 | AGATTAGATTTAAAAG TTTGAGTACACGTAAA |
| 49 | AGGCGGTCATTAGTCT TTAATGCGCAATATTA |
| 50 | GAATGGCTAGTATTAA CACCGCCTCAACTAAT |
| 51 | CCGCCAGCCATTGCAA CAGGAAAAATATTTTT |
| 52 | CCCTCAGAACCGCCAC CCTCAGAACTGAGACT |
| 53 | CCTCAAGAATACATGG CTTTTGATAGAACCAC |
| 54 | TAAGCGTCGAAGGATT AGGATTAGTACCGCCA |
| 55 | CACCAGAGTTCGGTCA TAGCCCCCGCCAGCAA |
| 56 | TCGGCATTCCGCCGCC AGCATTGACGTTCCAG |
| 57 | AATCACCAAATAGAAA ATTCATATATAACGGA |
| 58 | TCACAATCGTAGCACC ATTACCATCGTTTTCA |
| 59 | ATACCCAAGATAACCC ACAAGAATAAACGATT |
| 60 | ATCAGAGAAAGAACTG GCATGATTTTATTTTG |
| 61 | TTTTGTTTAAGCCTTA AATCAAGAATCGAGAA |
| 62 | AGGTTTTGAACGTCAA AAATGAAAGCGCTAAT |
| 63 | CAAGCAAGACGCGCCT GTTTATCAAGAATCGC |
| 64 | AATGCAGACCGTTTTT ATTTTCATCTTGCGGG |
| 65 | CATATTTAGAAATACC GACCGTGTTACCTTTT |
| 66 | AATGGTTTACAACGCC AACATGTAGTTCAGCT |
| 67 | TAACCTCCATATGTGA GTGAATAAACAAAATC |
| 68 | AAATCAATGGCTTAGG TTGGGTTACTAAATTT |
| 69 | GCGCAGAGATATCAAA ATTATTTGACATTATC |
| 70 | AACCTACCGCGAATTA TTCATTTCCAGTACAT |
| 71 | ATTTTGCGTCTTTAGG AGCACTAAGCAACAGT |
| 72 | CTAAAATAGAACAAAG AAACCACCAGGGTTAG |
| 73 | GCCACGCTATACGTGG CACAGACAACGCTCAT |
| 74 | GCGTAAGAGAGAGCCA GCAGCAAAAAGGTTAT |
| 75 | GGAAATACCTACATTT TGACGCTCACCTGAAA |
| 76 | TATCACCGTACTCAGG AGGTTTAGCGGGGTTT |
| 77 | TGCTCAGTCAGTCTCT GAATTTACCAGGAGGT |
| 78 | GGAAAGCGACCAGGCG GATAAGTGAATAGGTG |
| 79 | TGAGGCAGGCGTCAGA CTGTAGCGTAGCAAGG |
| 80 | TGCCTTTAGTCAGACG ATTGGCCTGCCAGAAT |
| 81 | CCGGAAACACACCACG GAATAAGTAAGACTCC |
| 82 | ACGCAAAGGTCACCAA TGAAACCAATCAAGTT |
| 83 | TTATTACGGTCAGAGG GTAATTGAATAGCAGC |
| 84 | TGAACAAACAGTATGT TAGCAAACTAAAAGAA |
| 85 | CTTTACAGTTAGCGAA CCTCCCGACGTAGGAA |
| 86 | GAGGCGTTAGAGAATA ACATAAAAGAACACCC |
| 87 | TCATTACCCGACAATA AACAACATATTTAGGC |
| 88 | CCAGACGAGCGCCCAA TAGCAAGCAAGAACGC |
| 89 | AGAGGCATAATTTCAT CTTCTGACTATAACTA |
| 90 | TTTTAGTTTTTCGAGC CAGTAATAAATTCTGT |
| 91 | TATGTAAACCTTTTTT AATGGAAAAATTACCT |
| 92 | TTGAATTATGCTGATG CAAATCCACAAATATA |
| 93 | GAGCAAAAACTTCTGA ATAATGGAAGAAGGAG |
| 94 | TGGATTATGAAGATGA TGAAACAAAATTTCAT |
| 95 | CGGAATTATTGAAAGG AATTGAGGTGAAAAAT |
| 96 | ATCAACAGTCATCATA TTCCTGATTGATTGTT |
| 97 | CTAAAGCAAGATAGAA CCCTTCTGAATCGTCT |
| 98 | GCCAACAGTCACCTTG CTGAACCTGTTGGCAA |
| 99 | GAAATGGATTATTTAC ATTGGCAGACATTCTG |
| 100 | TTTT TATAAGTA TAGCCCGGCCGTCGAG |
| 101 | AGGGTTGA TTTT ATAAATCC TCATTAAATGATATTC |
| 102 | ACAAACAA TTTT AATCAGTA GCGACAGATCGATAGC |
| 103 | AGCACCGT TTTT TAAAGGTG GCAACATAGTAGAAAA |
| 104 | TACATACA TTTT GACGGGAG AATTAACTACAGGGAA |
| 105 | GCGCATTA TTTT GCTTATCC GGTATTCTAAATCAGA |
| 106 | TATAGAAG TTTT CGACAAAA GGTAAAGTAGAGAATA |
| 107 | TAAAGTAC TTTT CGCGAGAA AACTTTTTATCGCAAG |
| 108 | ACAAAGAA TTTT ATTAATTA CATTTAACACATCAAG |
| 109 | AAAACAAA TTTT TTCATCAA TATAATCCTATCAGAT |
| 110 | GATGGCAA TTTT AATCAATA TCTGGTCACAAATATC |
| 111 | AAACCCTC TTTT ACCAGTAA TAAAAGGGATTCACCA GTCACACG TTTT |
| 112 | CCGAAATCCGAAAATC CTGTTTGAAGCCGGAA |
| 113 | CCAGCAGGGGCAAAAT CCCTTATAAAGCCGGC |
| 114 | GCATAAAGTTCCACAC AACATACGAAGCGCCA |
| 115 | GCTCACAATGTAAAGC CTGGGGTGGGTTTGCC |
| 116 | TTCGCCATTGCCGGAA ACCAGGCATTAAATCA |
| 117 | GCTTCTGGTCAGGCTG CGCAACTGTGTTATCC |
| 118 | GTTAAAATTTTAACCA ATAGGAACCCGGCACC |
| 119 | AGACAGTCATTCAAAA GGGTGAGAAGCTATAT |
| 120 | AGGTAAAGAAATCACC ATCAATATAATATTTT |
| 121 | TTTCATTTGGTCAATA ACCTGTTTATATCGCG |
| 122 | TCGCAAATGGGGCGCG AGCTGAAATAATGTGT |
| 123 | TTTTAATTGCCCGAAA GACTTCAAAACACTAT |
| 124 | AAGAGGAACGAGCTTC AAAGCGAAGATACATT |
| 125 | GGAATTACTCGTTTAC CAGACGACAAAAGATT |
| 126 | GAATAAGGACGTAACA AAGCTGCTCTAAAACA |
| 127 | CCAAATCACTTGCCCT GACGAGAACGCCAAAA |
| 128 | CTCATCTTGAGGCAAA AGAATACAGTGAATTT |
| 129 | AAACGAAATGACCCCC AGCGATTATTCATTAC |
| 130 | CTTAAACATCAGCTTG CTTTCGAGCGTAACAC |
| 131 | TCGGTTTAGCTTGATA CCGATAGTCCAACCTA |
| 132 | TGAGTTTCGTCACCAG TACAAACTTAATTGTA |
| 133 | CCCCGATTTAGAGCTT GACGGGGAAATCAAAA |
| 134 | GAATAGCCGCAAGCGG TCCACGCTCCTAATGA |
| 135 | GAGTTGCACGAGATAG GGTTGAGTAAGGGAGC |
| 136 | GTGAGCTAGTTTCCTG TGTGAAATTTGGGAAG |
| 137 | TCATAGCTACTCACAT TAATTGCGCCCTGAGA |
| 138 | GGCGATCGCACTCCAG CCAGCTTTGCCATCAA |
| 139 | GAAGATCGGTGCGGGC CTCTTCGCAATCATGG |
| 140 | AAATAATTTTAAATTG TAAACGTTGATATTCA |
| 141 | GCAAATATCGCGTCTG GCCTTCCTGGCCTCAG |
| 142 | ACCGTTCTAAATGCAA TGCCTGAGAGGTGGCA |
| 143 | TATATTTTAGCTGATA AATTAATGTTGTATAA |
| 144 | TCAATTCTTTTAGTTT GACCATTACCAGACCG |
| 145 | CGAGTAGAACTAATAG TAGTAGCAAACCCTCA |
| 146 | GAAGCAAAAAAGCGGA TTGCATCAGATAAAAA |
| 147 | TCAGAAGCCTCCAACA GGTCAGGATCTGCGAA |
| 148 | CCAAAATATAATGCAG ATACATAAACACCAGA |
| 149 | CATTCAACGCGAGAGG CTTTTGCATATTATAG |
| 150 | ACGAGTAGTGACAAGA ACCGGATATACCAAGC |
| 151 | AGTAATCTTAAATTGG GCTTGAGAGAATACCA |
| 152 | GCGAAACATGCCACTA CGAAGGCATGCGCCGA |
| 153 | ATACGTAAAAGTACAA CGGAGATTTCATCAAG |
| 154 | CAATGACACTCCAAAA GGAGCCTTACAACGCC |
| 155 | AAAAAAGGACAACCAT CGCCCACGCGGGTAAA |
| 156 | TGTAGCATTCCACAGA CAGCCCTCATCTCCAA |
| 157 | GTAAAGCACTAAATCG GAACCCTAGTTGTTCC |
| 158 | AGTTTGGAGCCCTTCA CCGCCTGGTTGCGCTC |
| 159 | AGCTGATTACAAGAGT CCACTATTGAGGTGCC |
| 160 | ACTGCCCGCCGAGCTC GAATTCGTTATTACGC |
| 161 | CCCGGGTACTTTCCAG TCGGGAAACGGGCAAC |
| 162 | CAGCTGGCGGACGACG ACAGTATCGTAGCCAG |
| 163 | GTTTGAGGGAAAGGGG GATGTGCTAGAGGATC |
| 164 | CTTTCATCCCCAAAAA CAGGAAGACCGGAGAG |
| 165 | AGAAAAGCAACATTAA ATGTGAGCATCTGCCA |
| 166 | GGTAGCTAGGATAAAA ATTTTTAGTTAACATC |
| 167 | CAACGCAATTTTTGAG AGATCTACTGATAATC |
| 168 | CAATAAATACAGTTGA TTCCCAATTTAGAGAG |
| 169 | TCCATATACATACAGG CAAGGCAACTTTATTT |
| 170 | TACCTTTAAGGTCTTT ACCCTGACAAAGAAGT |
| 171 | CAAAAATCATTGCTCC TTTTGATAAGTTTCAT |
| 172 | TTTGCCAGATCAGTTG AGATTTAGTGGTTTAA |
| 173 | AAAGATTCAGGGGGTA ATAGTAAACCATAAAT |
| 174 | TTTCAACTATAGGCTG GCTGACCTTGTATCAT |
| 175 | CCAGGCGCTTAATCAT TGTGAATTACAGGTAG |
| 176 | CGCCTGATGGAAGTTT CCATTAAACATAACCG |
| 177 | TTTCATGAAAATTGTG TCGAAATCTGTACAGA |
| 178 | ATATATTCTTTTTTCA CGTTGAAAATAGTTAG |
| 179 | AATAATAAGGTCGCTG AGGCTTGCAAAGACTT |
| 180 | CGTAACGATCTAAAGT TTTGTCGTGAATTGCG |
| 181 | ACCCAAATCAAGTTTT TTGGGGTCAAAGAACG |
| 182 | TGGACTCCCTTTTCAC CAGTGAGACCTGTCGT |
| 183 | TGGTTTTTAACGTCAA AGGGCGAAGAACCATC |
| 184 | GCCAGCTGCCTGCAGG TCGACTCTGCAAGGCG |
| 185 | CTTGCATGCATTAATG AATCGGCCCGCCAGGG |
| 186 | ATTAAGTTCGCATCGT AACCGTGCGAGTAACA |
| 187 | TAGATGGGGGGTAACG CCAGGGTTGTGCCAAG |
| 188 | ACCCGTCGTCATATGT ACCCCGGTAAAGGCTA |
| 189 | CATGTCAAGATTCTCC GTGGGAACCGTTGGTG |
| 190 | TCAGGTCACTTTTGCG GGAGAAGCAGAATTAG |
| 191 | CTGTAATATTGCCTGA GAGTCTGGAAAACTAG |
| 192 | CAAAATTAAAGTACGG TGTCTGGAAGAGGTCA |
| 193 | TGCAACTAAGCAATAA AGCCTCAGTTATGACC |
| 194 | TTTTTGCGCAGAAAAC GAGAATGAATGTTTAG |
| 195 | AAACAGTTGATGGCTT AGAGCTTATTTAAATA |
| 196 | ACTGGATAACGGAACA ACATTATTACCTTATG |
| 197 | ACGAACTAGCGTCCAA TACTGCGGAATGCTTT |
| 198 | CGATTTTAGAGGACAG ATGAACGGCGCGACCT |
| 199 | CTTTGAAAAGAACTGG CTCATTATTTAATAAA |
| 200 | GCTCCATGAGAGGCTT TGAGGACTAGGGAGTT |
| 201 | ACGGCTACTTACTTAG CCGGAACGCTGACCAA |
| 202 | AAAGGCCGAAAGGAAC AACTAAAGCTTTCCAG |
| 203 | GAGAATAGCTTTTGCG GGATCGTCGGGTAGCA |
| 204 | ACGTTAGTAAATGAAT TTTCTGTAAGCGGAGT |
| 205 | TTTT CGATGGCC CACTACGTAAACCGTC |
| 206 | TATCAGGG TTTT CGGTTTGC GTATTGGGAACGCGCG |
| 207 | GGGAGAGG TTTT TGTAAAAC GACGGCCATTCCCAGT |
| 208 | CACGACGT TTTT GTAATGGG ATAGGTCAAAACGGCG |
| 209 | GATTGACC TTTT GATGAACG GTAATCGTAGCAAACA |
| 210 | AGAGAATC TTTT GGTTGTAC CAAAAACAAGCATAAA |
| 211 | GCTAAATC TTTT CTGTAGCT CAACATGTATTGCTGA |
| 212 | ATATAATG TTTT CATTGAAT CCCCCTCAAATCGTCA |
| 213 | TAAATATT TTTT GGAAGAAA AATCTACGACCAGTCA |
| 214 | GGACGTTG TTTT TCATAAGG GAACCGAAAGGCGCAG |
| 215 | ACGGTCAA TTTT GACAGCAT CGGAACGAACCCTCAG |
| 216 | CAGCGAAAA TTTT ACTTTCA ACAGTTT CTGGGA TTTTGCT AAACTTTT |

| Table S2. The hybridizing with AgNPs/Si sequences and modification sites (left to right 5’-3’)**.** | |
| --- | --- |
| Name | DNA Sequence |
| 146 | GAAGCAAAAAAGCGGATTGCATCAGATAAAAA  +TTTTT+polyA_40_ |
| 124 | AAGAGGAACGAGCTTCAAAGCGAAGATACATT+TTTTT+polyA_40_ |
| 123 | TTTTAATTGCCCGAAAGACTTCAAAACACTAT+TTTTT+polyA_40_ |
| 11 | CCTAATTTACGCTAACGAGCGTCTAATCAATA+TTTTT+polyA_40_ |
| 12 | TCTTACCAGCCAGTTACAAAATAAATGAAATA+TTTTT+polyA_40_ |
| 37 | ATTATTTAACCCAGCTACAATTTTCAAGAACG+TTTTT+polyA_40_ |
| 142 | ACCGTTCTAAATGCAATGCCTGAGAGGTGGCA+TTTTT+polyA_40_ |
| 120 | AGGTAAAGAAATCACCATCAATATAATATTTT+TTTTT+  polyA_40_ |
| 119 | AGACAGTCATTCAAAAGGGTGAGAAGCTATAT+TTTTT+polyA_40_ |
| 15 | GCGTTATAGAAAAAGCCTGTTTAGAAGGCCGG+TTTTT+polyA_40_ |
| 17 | AATTACTACAAATTCTTACCAGTAATCCCATC+TTTTT+  polyA_40_ |
| 41 | ACGCTCAAAATAAGAATAAACACCGTGAATTT+TTTTT+polyA_40_ |

| Table S3. The IL-6 detection sequences and modification sites (left to right 5’-3’)**.** | |
| --- | --- |
| Name | DNA Sequence |
| 200 | GCTCCATGAGAGGCTTTGAGGACTAGGGAGTT+TTTTT+CTTCCAACGCTCGTATTCTAGTCTTTAGT+Cy3 |
| 152 | GCGAAACATGCCACTACGAAGGCATGCGCCGA+TTTTT+CTTCCAACGCTCGTATTCTAGTCTTTAGT+Cy3 |
| 5 | AACCAGAGACCCTCAGAACCGCCAGGGGTCAG+TTTTT+CTTCCAACGCTCGTATTCTAGTCTTTAGT+Cy3 |
| 56 | TCGGCATTCCGCCGCCAGCATTGACGTTCCAG+TTTTT+CTTCCAACGCTCGTATTCTAGTCTTTAGT+Cy3 |
| 192 | CAAAATTAAAGTACGGTGTCTGGAAGAGGTCA+TTTTT+CTTCCAACGCTCGTATTCTAGTCTTTAGT+Cy3 |
| 144 | TCAATTCTTTTAGTTTGACCATTACCAGACCG+TTTTT+CCTTCCAACGCTCGTATTCTAGTCTTTAGT+Cy3 |
| 14 | CTAATTTATCTTTCCTTATCATTCATCCTGAA+TTTTT+CATCTTCCAACGCTCGTATTCTAGTCTTTAGT+Cy3 |
| 64 | AATGCAGACCGTTTTTATTTTCATCTTGCGGG+TTTTT+CCTTCCAACGCTCGTATTCTAGTCTTTAGT+Cy3 |
| 184 | GCCAGCTGCCTGCAGGTCGACTCTGCAAGGCG+TTTTT+CTTCCAACGCTCGTATTCTAGTCTTTAGT+Cy3 |
| 136 | GTGAGCTAGTTTCCTGTGTGAAATTTGGGAAG+TTTTT+CTTCCAACGCTCGTATTCTAGTCTTTAGT+Cy3 |
| 23 | GGATTTAGCGTATTAAATCCTTTGTTTTCAGG+TTTTT+CTTCCAACGCTCGTATTCTAGTCTTTAGT+Cy3 |
| 72 | CTAAAATAGAACAAAGAAACCACCAGGGTTAG+TTTTT+CTTCCAACGCTCGTATTCTAGTCTTTAGT+Cy3 |
| IL-6 aptamers | AAATACGAGCGTTGGAAGTA+ROX |

Table S4. The assignment of Raman characteristic peaks**.**

| Vibrational description | Cy3 | | |
| --- | --- | --- | --- |
|  | Observed | | Reported |
| v(C-H)_ip-bend_ | 1192 | | 1193 |
| v(C=C)_ring-stretch_ | 1466 | | 1465 |
| v(C=N)_stretch_ | 1595 | | 1586 |
|  | ROX | | |
| v(C-C)_ring-stretch_ | 1350 | 1344 | |
| v(C-C)_ring-stretch_ | 1507 | 1499 | |
| v(C-C)_ring-stretch_ | 1649 | 1644 | |

Table S5. The Ca^2+^ detection sequences and modification sites (left to right 5’-3’)**.**

| **Ca^2+^ detection** | |
| --- | --- |
| Name | DNA Sequence |
| 200 | GCTCCATGAGAGGCTTTGAGGACTAGGGAGTT+TTTTT+ TTTCGCCATCTTTTCTCACAGCGTACTCGCTAAGGTTGTTAGTGACTCGTGAC+Cy3 |
| 152 | GCGAAACATGCCACTACGAAGGCATGCGCCGA+TTTTT+ TTTCGCCATCTTTTCTCACAGCGTACTCGCTAAGGTTGTTAGTGACTCGTGAC+Cy3 |
| 5 | AACCAGAGACCCTCAGAACCGCCAGGGGTCAG+TTTT+TTTCGCCATCTTTTCTCACAGCGTACTCGCTAAGGTTGTTAGTGACTCGTGAC+Cy3 |
| 56 | TCGGCATTCCGCCGCCAGCATTGACGTTCCAG+TTTTT+TTTCGCCATCTTTTCTCACAGCGTACTCGCTAAGGTTGTTAGTGACTCGTGAC+Cy3 |
| 192 | CAAAATTAAAGTACGGTGTCTGGAAGAGGTCA+TTTTT+TTTCGCCATCTTTTCTCACAGCGTACTCGCTAAGGTTGTTAGTGACTCGTGAC+Cy3 |
| 144 | TCAATTCTTTTAGTTTGACCATTACCAGACCG+TTTTT+TTTCGCCATCTTTTCTCACAGCGTACTCGCTAAGGTTGTTAGTGACTCGTGAC+Cy3 |
| 14 | CTAATTTATCTTTCCTTATCATTCATCCTGAA+TTTTT+TTTCGCCATCTTTTCTCACAGCGTACTCGCTAAGGTTGTTAGTGACTCGTGAC+Cy3 |
| 64 | AATGCAGACCGTTTTTATTTTCATCTTGCGGG+TTTTT+TTTCGCCATCTTTTCTCACAGCGTACTCGCTAAGGTTGTTAGTGACTCGTGAC+Cy3 |
| 184 | GCCAGCTGCCTGCAGGTCGACTCTGCAAGGCG+TTTTT+TTTCGCCATCTTTTCTCACAGCGTACTCGCTAAGGTTGTTAGTGACTCGTGAC+Cy3 |
| 136 | GTGAGCTAGTTTCCTGTGTGAAATTTGGGAAG+TTTTT+TTTCGCCATCTTTTCTCACAGCGTACTCGCTAAGGTTGTTAGTGACTCGTGAC+Cy3 |
| 23 | GGATTTAGCGTATTAAATCCTTTGTTTTCAGG+TTTTT+TTTCGCCATCTTTTCTCACAGCGTACTCGCTAAGGTTGTTAGTGACTCGTGAC+Cy3 |
| 72 | CTAAAATAGAACAAAGAAACCACCAGGGTTAG+TTTTT+TTTCGCCATCTTTTCTCACAGCGTACTCGCTAAGGTTGTTAGTGACTCGTGAC+Cy3 |
| Ca^2+^ aptamers | GTCACGAGTCACTATrAGGAAGATGGCGAAA+ROX |

| Table S6. The miRNA-214 detection sequences and modification sites (left to right 5’-3’)**.** | |
| --- | --- |
| Name | DNA Sequence |
| 200 | GCTCCATGAGAGGCTTTGAGGACTAGGGAGTT+TTTTT+ AGCAGGGGACGACCCCCCTCGTCCCGGCAGT +Cy3 |
| 152 | GCGAAACATGCCACTACGAAGGCATGCGCCGA+TTTTT+ AGCAGGGGACGACCCCCCTCGTCCCGGCAGT +Cy3 |
| 5 | AACCAGAGACCCTCAGAACCGCCAGGGGTCAG+TTTTT+ AGCAGGGGACGACCCCCCTCGTCCCGGCAGT +Cy3 |
| 56 | TCGGCATTCCGCCGCCAGCATTGACGTTCCAG+TTTTT+ AGCAGGGGACGACCCCCCTCGTCCCGGCAGT +Cy3 |
| 192 | CAAAATTAAAGTACGGTGTCTGGAAGAGGTCA+TTTTT+ AGCAGGGGACGACCCCCCTCGTCCCGGCAGT +Cy3 |
| 144 | TCAATTCTTTTAGTTTGACCATTACCAGACCG+TTTTT+AGCAGGGGACGACCCCCCTCGTCCCGGCAGT +Cy3 |
| 14 | CTAATTTATCTTTCCTTATCATTCATCCTGAA+TTTTT+AGCAGGGGACGACCCCCCTCGTCCCGGCAGT +Cy3 |
| 64 | AATGCAGACCGTTTTTATTTTCATCTTGCGGG+TTTTT+AGCAGGGGACGACCCCCCTCGTCCCGGCAGT +Cy3 |
| 184 | GCCAGCTGCCTGCAGGTCGACTCTGCAAGGCG+TTTTT+ AGCAGGGGACGACCCCCCTCGTCCCGGCAGT +Cy3 |
| 136 | GTGAGCTAGTTTCCTGTGTGAAATTTGGGAAG+TTTTT+ AGCAGGGGACGACCCCCCTCGTCCCGGCAGT +Cy3 |
| 23 | GGATTTAGCGTATTAAATCCTTTGTTTTCAGG+TTTTT+ AGCAGGGGACGACCCCCCTCGTCCCGGCAGT +Cy3 |
| 72 | CTAAAATAGAACAAAGAAACCACCAGGGTTAG+TTTTT+ AGCAGGGGACGACCCCCCTCGTCCCGGCAGT+Cy3 |
| miRNA-214 aptamers | ACTGCCTGTCTGTGCCTGCTGT+ROX |
| miRNA-214 | ACAGCAGGCACAGACAGGCAGU |

**Table S7. Comparative evaluation of analytical methods for biomarker detection**

| Analytical methods | Features | | | | | |
| --- | --- | --- | --- | --- | --- | --- |
|  | Sample pretreatment | Equipment complexity | On-site Applicability | Total assay time | Professional Requirement | Extreme Environments |
| ELISA | Simple | High | No | >2 h | Yes | Poor |
| qPCR | Simple | High | No | >2 h | Yes | Poor |
| MS | Complex | \| Very high \| \| --- \| | No | >2 h | Yes | Very poor |
| **This work** | **Simple** | **Low** | **Yes** | **20 min** | **No** | **Excellent** |

**Table S8. CNN network classification report**

|  | Precision | Recall | F1-score | Support |
| --- | --- | --- | --- | --- |
| 0(Healthy) | 0.925 | 0.974 | 0.949 | 38 |
| 1(Healthy) | 0.986 | 0.96 | 0.973 | 75 |
| Accuracy | 0.965 | 0.965 | 0.965 | 0.965 |
| Macro avg | 0.956 | 0.967 | 0.961 | 113 |
| Weighted avg | 0.966 | 0.965 | 0.965 | 113 |
